# Do Acceptance‐ and Mindfulness‐Based Interventions Improve Psychological Flexibility in People With Chronic Pain? A Systematic Review and Meta‐Analysis of Randomized Controlled Trials

**DOI:** 10.1002/ejp.70342

**Published:** 2026-08-01

**Authors:** Juan P. Sanabria‐Mazo, Carla Rodríguez‐Freire, Estíbaliz Royuela‐Colomer, Paula Ponce‐López, Pablo Alonso‐Coello, Valentina Barrios, Jaime Navarrete, Adrián Pérez‐Aranda, Lance M. McCracken, Juan V. Luciano

**Affiliations:** ^1^ Unit for the Study and Treatment of Pain—ALGOS, Department of Psychology, Research Center for Behavior Assessment (CRAMC) Universitat Rovira i Virgili Tarragona Spain; ^2^ Institut d'investigació Sanitària Pere Virgili Universitat Rovira i Virgili Tarragona Spain; ^3^ Teaching, Research & Innovation Unit Parc Sanitari Sant Joan de Déu St. Boi de Llobregat Spain; ^4^ Department of Clinical and Health Psychology Autonomous University of Barcelona Cerdanyola del Vallès Spain; ^5^ Centre for Biomedical Research in Epidemiology and Public Health (CIBERESP) Madrid Spain; ^6^ Iberoamerican Cochrane Centre Biomedical Research Institute Sant Pau (IIB SantPau) Barcelona Spain; ^7^ Research Institute‐Faculty of Psychology University of Buenos Aires Buenos Aires Argentina; ^8^ Psychology Department Uppsala University Uppsala Sweden

## Abstract

**Background:**

Acceptance‐ and mindfulness‐based interventions are effective for improving pain‐related outcomes, yet their effects on psychological flexibility have not been meta‐analyzed. This systematic review and meta‐analysis addressed this gap by comparing these interventions with control conditions on psychological flexibility and its core dimensions in adults with chronic pain.

**Methods:**

Searches were conducted in PsycINFO, MEDLINE, Cochrane CENTRAL, Scopus and Web of Science from inception to May 2025. Sixty randomized controlled trials comprising 6692 participants were included. Meta‐analyses were conducted for global psychological flexibility (*k* = 17), acceptance (*k* = 38), committed action (*k* = 4) and values (*k* = 4). Heterogeneity, risk of bias and certainty of evidence were assessed.

**Results:**

Interventions significantly improved psychological flexibility at post‐treatment, short‐term (≤ 6 months) and long‐term (> 6 months) follow‐up (*g* = 0.35–0.69), and acceptance across all time points (*g* = 0.41–0.77). Effects for committed action and values were non‐significant. Heterogeneity was substantial, most trials had a high risk of bias, and the certainty of evidence was low to very low. Moderator analyses indicated larger effects when interventions were compared with inactive controls, delivered by psychologists or implemented as stand‐alone programs.

**Conclusions:**

Evidence suggests that acceptance‐ and mindfulness‐based interventions may improve global psychological flexibility and acceptance. However, the certainty of evidence was low to very low, and most included trials were at high risk of bias. More rigorous trials are needed to clarify effects across specific dimensions of psychological flexibility.

**Significance Statement:**

This systematic review and meta‐analysis provide the first comprehensive synthesis of the effects of acceptance‐ and mindfulness‐based interventions on psychological flexibility in chronic pain. These findings show that these interventions improve psychological flexibility, particularly pain acceptance, supporting it as a clinically relevant treatment target.

**Trial Registration:**

PROSPERO: CRD420251018441

## 1 Introduction

Chronic pain is a prevalent condition associated with impairments in daily functioning and psychological wellbeing (Cohen et al. 2021; Rometsch et al. 2025). Cognitive behavioural therapy (CBT) remains the most established psychological intervention for chronic pain, although its overall effects are relatively modest (Williams et al. 2020). Building on this tradition, contextual cognitive‐behavioural approaches place greater emphasis on acceptance and mindfulness and have shown beneficial effects across pain‐related outcomes (McCracken et al. 2022).

Meta‐analytic evidence indicates that acceptance‐ and mindfulness‐based interventions (MBIs) improve pain interference, pain intensity, depression and anxiety (Veehof et al. 2016). Specifically, acceptance and commitment therapy (ACT) shows strong effects on pain interference (Lai et al. 2023; Ma et al. 2023), whereas MBIs show moderate effects on pain interference, depression and quality of life (Hilton et al. 2017; Paschali et al. 2024). However, available syntheses have focused primarily on outcomes rather than on whether these interventions improve the psychological processes they are designed to target.

One such process is psychological flexibility, defined as the capacity to remain open to aversive internal experiences while acting in line with personal values (Hayes et al. 2012). The hexaflex model describes six interrelated dimensions of this construct: acceptance, cognitive defusion, present‐moment awareness, self‐as‐context, values and committed action (Scott et al. 2016). In chronic pain, psychological flexibility captures adaptive responding to pain and distress (McCracken 2024) and has been examined as a mediator of treatment effects (Pérez‐Aranda et al. 2019; Scott et al. 2016; Vowles et al. 2014; Wicksell et al. 2010). Nonetheless, the assessment of psychological flexibility remains debated, particularly regarding whether it is best represented by global, multidimensional or process‐specific measures (Lucas et al. 2025), and some dimensions, such as cognitive defusion, present‐moment awareness and self‐as‐context, appear to be examined less in chronic pain intervention research.

Despite substantial evidence supporting psychological flexibility as a therapeutic process in chronic pain (McCracken and Morley 2014; McCracken and Vowles 2014), to our knowledge, no meta‐analysis has yet examined whether acceptance‐ and MBIs improve psychological flexibility itself and its dimensions. Meta‐analyses of intervention studies have primarily focused on outcomes (Hilton et al. 2017; Lai et al. 2023; Paschali et al. 2024; Veehof et al. 2016), whereas meta‐analyses of psychological flexibility have examined its association with pain‐related outcomes (Ding and Zheng 2022; Fang and Ding 2022).

This systematic review and meta‐analysis address this gap by examining the effects of acceptance‐ and MBIs on psychological flexibility and its dimensions in people with chronic pain. Potential moderators of these effects were also examined, including control condition type, intervention type, delivery mode, treatment format, therapist involvement, pain diagnosis, intervention provider, intervention structure, instrument type, intervention duration, session duration, session frequency, dropout rate, proportion of females and age. Based on the theoretical focus of these interventions (McCracken et al. 2022), we expected significant improvements in global psychological flexibility and acceptance. Given the anticipated clinical and methodological heterogeneity across randomized controlled trials (RCTs), we expected larger effects in trials using inactive comparators, stand‐alone interventions and psychologist‐delivered interventions.

## 2 Methods

### 2.1 Study Design

This study followed the Preferred Reporting Items for Systematic Reviews and Meta‐Analyses (PRISMA) guidelines (Page et al. 2021). Methodological standards were based on the Cochrane Handbook for Systematic Reviews of Interventions (Higgins et al. 2024) and A Measurement Tool to Assess Systematic Reviews 2 (AMSTAR 2) (Shea et al. 2017). The review protocol was registered in the International Prospective Register of Systematic Reviews (PROSPERO).

### 2.2 Data Sources

A search strategy was conducted using PsycINFO (ProQuest), Medline (PubMed), Cochrane Central Register of Controlled Trials (CENTRAL), Scopus (Elsevier) and Web of Science (Core Collection). Additionally, a complementary search was conducted in the Association for Contextual Behavioural Science (ACBS) registry of ACT RCTs from 1986 to the present. The general search strategy combined terms related to “Population” (chronic pain), “Intervention” (acceptance‐ and MBIs) and “Outcome” (psychological [in]flexibility), encompassing both the global construct and its six core dimensions (acceptance or experiential avoidance, cognitive defusion or cognitive fusion, present‐moment awareness or lack of present‐moment awareness, self‐as‐context or self‐as‐content, values or lack of values and committed action or inaction). For simplicity, the term psychological (in)flexibility will hereafter be referred to as psychological flexibility, and all dimensions will be presented in their flexible form.

### 2.3 Search Strategy

The search strategy combined free‐text terms with the American Psychological Association Thesaurus and Medical Subject Headings, using Boolean operators adapted to each database. Searches were conducted across titles, abstracts, keywords and subject headings to maximize retrieval. Search terms were informed by strategies used in previous reviews on acceptance‐ and MBIs (Hilton et al. 2017; Lai et al. 2023; Martinez‐Calderon et al. 2024; Sanabria‐Mazo, Colomer‐Carbonell, Fernández‐Vázquez, et al. 2023; Veehof et al. 2016; Williams et al. 2020) and on psychological flexibility (Ding and Zheng 2022; Fang and Ding 2022) in chronic pain populations. The final search strategy was refined in accordance with the Peer Review of Electronic Search Strategies (PRESS) guidelines (McGowan et al. 2016). In addition, reference lists of included studies and published reviews on related topics were screened to identify further eligible trials. The following filters were applied in all databases, where possible: (1) publication date (from inception until May 2025), (2) document type (peer‐reviewed articles only) and (3) languages (English, Spanish and French). Complete search strategies for each database are provided in Table S1.

### 2.4 Eligibility Criteria

The eligibility criteria were defined following the ‘Population’, ‘Intervention’, ‘Comparison’, ‘Outcomes’ and ‘Study design’ (PICOS) framework (Methley et al. 2014). The specific selection criteria applied in this study are detailed below.

#### 2.4.1 [P] Population

Adults (≥ 18 years) with non‐oncologic chronic pain (> 3 months) were included. Individuals with cancer‐related pain, acute pain (< 3 months), postsurgical pain, pain of unspecified duration or headaches with distinct pathophysiological mechanisms, as well as those with comorbid severe psychiatric disorders (psychosis, eating disorders or conditions requiring hospitalization), substance dependence or neurodegenerative diseases, were excluded.

#### 2.4.2 [I] Intervention

Acceptance‐ and MBIs were included regardless of delivery mode (face‐to‐face, online or blended), timing (synchronous with real‐time therapist interaction or asynchronous self‐paced) or treatment format (individual or group). These interventions encompassed three main categories: ACT, MBI and contextual cognitive behavioural therapy (CCBT). MBI comprised standardized protocols, such as mindfulness‐based stress reduction (MBSR) and mindfulness‐based cognitive therapy (MBCT), as well as other mindfulness‐based programs. In contrast, CCBT referred to contextual approaches that incorporate acceptance‐ and/or mindfulness‐based processes but do not strictly follow ACT or MBI protocols. CCBTs were included only in the systematic review, whereas ACT and MBI were retained for meta‐analyses to preserve protocol homogeneity. Interventions were further classified as stand‐alone (acceptance‐ or mindfulness‐based only) or combined (integrated with other therapeutic elements) when acceptance‐ or mindfulness‐based processes were explicitly incorporated as core therapeutic components. Interventions in which pharmacotherapy constituted a primary component were excluded.

#### 2.4.3 [C] Comparison

Studies were included exclusively if they compared an acceptance‐ and mindfulness‐based intervention to an active (e.g., relaxation training, psychoeducation or physical therapy) or an inactive control condition (e.g., treatment as usual, waitlist or attention control). Multi‐arm trials were considered if at least one comparison met these criteria. Studies without a control group were excluded. In addition, RCTs directly comparing two acceptance‐ and MBIs (e.g., ACT vs. CCBT or MBI vs. CCBT) were included in the systematic review but excluded from the meta‐analysis.

#### 2.4.4 [O] Outcomes

Only studies that measured psychological flexibility as a global construct and/or at least one of its six core dimensions (i.e., acceptance, cognitive defusion, present‐moment awareness, self‐as‐context, values and committed action) using validated measures were retained (McCracken 2024). In this review, global psychological flexibility was defined as a broad measure intended to capture psychological flexibility or inflexibility beyond a single hexaflex dimension. This category included instruments such as the Psychological Inflexibility in Pain Scale (PIPS) and the Acceptance and Action Questionnaire‐II (AAQ‐II). This operationalization was adopted because these instruments are commonly used as broad indicators of psychological flexibility‐related processes in chronic pain research, despite ongoing debate about the dimensional structure of psychological flexibility and inflexibility (Lucas et al. 2025). Studies that did not report sufficient statistical data to estimate the intervention's effect on psychological flexibility were excluded from the meta‐analysis but were summarized narratively in the systematic review.

#### 2.4.5 [S] Study Design

Only parallel RCTs with ethical approval and publication in peer‐reviewed journals were eligible. Pilot studies were also eligible if they adopted a parallel RCT design. Crossover RCTs were excluded due to the potential risk of carryover effects on psychological flexibility outcomes. Other study designs were excluded.

### 2.5 Selection Process

Study selection was conducted using Rayyan QCRI (Ouzzani et al. 2016). First, duplicate records were automatically removed. Second, three reviewers (CR‐F, PP‐L and JN) independently and blindly screened titles and abstracts. When information was insufficient to determine eligibility, full texts were reviewed. All retrieved articles were assessed against the eligibility criteria, and relevant studies were selected by cross‐examination. Discrepancies were resolved by consensus, and those arising during full‐text screening were resolved with the involvement of an additional reviewer (JPS‐M). Finally, the references of the included articles and reviews identified through the search were screened to capture additional relevant studies not detected by the initial search strategies.

### 2.6 Data Extraction and Management

Data extraction was conducted independently by two reviewers (JPS‐M and VB) using a standardized extraction form adapted from the Cochrane guidelines (Higgins et al. 2024). Extracted data were cross‐checked for accuracy and reliability, and the corresponding authors were contacted to provide clarification when information required for the meta‐analysis was missing or incomplete. Two reminder emails were sent at two‐week intervals if no response was received. In cases where the means and standard deviations required to compute instrument scores were neither reported nor provided by the study authors, they were estimated from the available data (e.g., sample size, medians, ranges, interquartile ranges or subscale scores) according to the method proposed by Wan et al. (2014).

Data were collected across three categories: (1) study characteristics (first author, year of publication, country and total sample size); (2) sample characteristics (age, gender, pain diagnosis, years with pain and pain intensity); and (3) intervention and control characteristics (control condition type, intervention type, delivery mode, treatment format, therapist involvement, intervention provider, intervention structure, intervention duration, assessment points, dropout rates, outcomes, measurement instruments and outcome role).

### 2.7 Risk of Bias and Certainty of the Evidence

The risk of bias at the study level and the certainty of the evidence at the outcome level were independently assessed by two reviewers (ER‐C and CR‐F) using the Revised Cochrane Risk‐of‐Bias Tool for Randomized Trials (RoB 2) (Sterne et al. 2019) and the Grading of Recommendations Assessment, Development and Evaluation (GRADE) approach (Schünemann et al. 2013), respectively. RoB Figures were created using the Risk‐Of‐Bias VISualization (Robvis) package in R (McGuinness 2021; McGuinness and Higgins 2020). Discrepancies were resolved with the involvement of an additional reviewer (JPS‐M).

RoB 2 evaluates potential bias across five domains: (1) bias arising from the randomization process, (2) bias due to deviations from intended interventions, (3) bias due to missing outcome data, (4) bias in outcome measurement and (5) bias in the selection of reported results. Each domain consists of a series of questions with responses categorized as ‘yes’, ‘probably yes’, ‘probably no’, ‘no’ and ‘no information’. An algorithm then generates a judgement for each domain, classifying the risk of bias as ‘low risk’, ‘some concerns’ or ‘high risk’.

The GRADE approach was used to assess the certainty of the evidence for each outcome across four levels: ‘high’, ‘moderate’, ‘low’ or ‘very low’. Assessments were based on five domains: (1) risk of bias, (2) inconsistency, (3) indirectness, (4) imprecision and (5) publication bias. Following established operational criteria (Ryan and Hill 2016), evidence was downgraded or upgraded as appropriate.

### 2.8 Data Synthesis and Analysis

A systematic review was conducted to provide a descriptive synthesis of the studies. Study, sample, intervention and control characteristics were summarized narratively and tabulated to capture variability across RCTs. Subsequently, meta‐analyses were performed using the *metafor* and *dmetar* (Companion R Package for the Guide Doing Meta‐Analysis in R 2019; Viechtbauer 2010) packages in R to evaluate the effectiveness of acceptance‐ and MBIs in improving psychological flexibility and its dimensions compared to active or inactive control conditions. To ensure robustness, a minimum of three effect sizes was required for inclusion in each meta‐analysis (Cheung 2019).

Effect sizes were estimated using Hedges' *g*, calculated from post‐treatment scores, with statistical significance set at *p* = 0.05 and magnitudes classified as trivial (< 0.20), small (0.20–0.49), medium (0.50–0.79), large (0.80–1.19) and very large (≥ 1.20). For consistency, all effect sizes were coded such that higher scores indicated greater psychological flexibility. Accordingly, effect sizes derived from measures of inflexibility (e.g., PIPS or AAQ‐II) were multiplied by −1. Following Cochrane guidelines (Higgins et al. 2024), different versions of the same instrument were combined when psychometric evidence supported their equivalence, as they were considered to assess the same underlying construct. Distinct validated instruments measuring the same construct were included in the meta‐analyses when their conceptual and psychometric comparability had been established in previous research (McCracken 2024).

For studies reporting multiple post‐treatment assessments at different time points, the following approach was applied: (1) the first post‐treatment assessment was used to represent the immediate effects of the intervention; (2) follow‐up assessments conducted within six months after baseline, excluding the first post‐treatment assessment, were averaged to estimate short‐term effects; and (3) follow‐up assessments conducted beyond six months from baseline were averaged to estimate long‐term effects. Averages were required in only three studies, as most assessments aligned with predefined time points (post, ≤ 6 months and > 6 months) (Higgins et al. 2024). In multi‐arm trials, intervention or control groups within the same category were statistically pooled following Cochrane guidelines (Higgins et al. 2024) to generate a single effect size per comparison, using the corresponding formulas for pooled means and standard deviations. The groups were merged using the function provided by Harrer et al. (2019) in R. The specific cases where this approach was applied are detailed in Section 3.

Random‐effects models were applied to pool effect sizes (Harrer et al. 2019). Heterogeneity was assessed using Cochran's *Q* test and the *I* ^2^ statistic, with *I* ^2^ values categorized as low (< 30%), moderate (30%–75%) and high (> 75%). Subgroup analyses were performed for categorical moderators, including control condition type (inactive or active), intervention type (ACT or MBI), delivery mode (face‐to‐face, internet‐based or blended/other), treatment format (individual, group or combined), therapist involvement (therapist‐delivered, self‐guided or combined), pain diagnosis (unspecified chronic pain, localized musculoskeletal pain and fibromyalgia and other widespread pain), intervention provider (psychologist, non‐psychologist or none), intervention structure (stand‐alone or combined) and instrument type (PIPS, AAQ‐II or other). For the subgroup analysis, a mixed‐effects model was used. When the number of studies in a subgroup was *k* ≤ 5, a pooled estimate of *τ2* (tau‐squared) was calculated (Borenstein et al. 2009). As recommended by Harrer et al. (2019), subgroup analyses were conducted only when heterogeneity was statistically significant (*p* < 0.10) and at least 10 studies were available for the overall moderator analysis.

Additionally, meta‐regression analyses were computed for continuous moderators using a weighted least squares (WLS) approach, which assigns greater weight to studies with smaller standard errors. The analyses followed the method of moments procedure with Knapp–Hartung correction to improve the robustness of estimates and included intervention duration (weeks), session duration (minutes), session frequency (number of sessions), dropout rates (%), proportion of females (%) and age (years). Publication bias was assessed through funnel plots and Egger's regression intercept to evaluate potential asymmetry in effect size distribution.

## 3 Results

### 3.1 Selection of Studies

The initial search identified a total of 3528 records. After duplicates were removed and screening was completed, 100 full‐text articles were assessed for eligibility. Of these, 40 records were excluded for the following reasons: outcome measures (*k* = 19), study design (*k* = 12), full text not available (*k* = 3), population outside the target scope (*k* = 2), publication types not eligible (*k* = 2) and insufficient or unclear data (*k* = 2). An additional eight articles were identified through reference list screening (Buhler et al. 2021; Buhrman et al. 2015; Clarke et al. 2017; Godfrey et al. 2020; Liu et al. 2025; Morone et al. 2008; Plumb et al. 2022; Thorsell et al. 2011). No additional records were added through other sources. The records excluded during the full‐text assessment are listed in Table S2.

**FIGURE 1:**
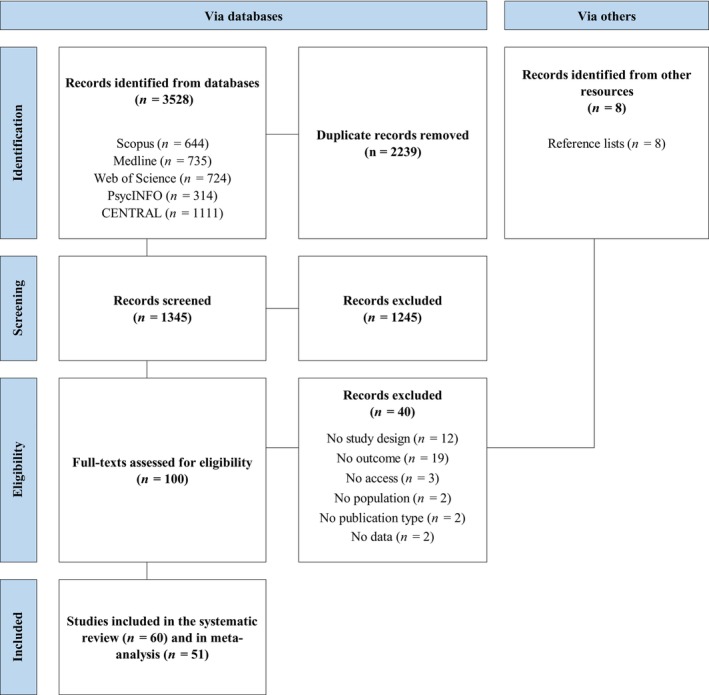
PRISMA flow diagram of included studies.

Some RCTs were reported in more than one publication but were treated as single studies in this systematic review: Wicksell et al. (2008, 2010), Thorsell et al. (2011, 2016), Kristjánsdóttir et al. (2013a, 2013b) and Casey et al. (2022, 2024). In addition, nine studies were excluded from the meta‐analysis for the following reasons: Wetherell et al. (2011), because it directly compared ACT with CCBT; Herbert et al. (2017), because it compared two ACT delivery formats without a control group; Godfrey et al. (2020), because essential statistical information was unavailable (despite two reminder emails); Torrijos‐Zarcero et al. (2021), because it compared MBI with CCBT; and Buhrman et al. (2015), Pincus et al. (2015), Gasslander et al. (2022), Bostrøm et al. (2023) and Solberg Nes et al. (2024), because they tested CCBT that incorporates acceptance‐ and/or mindfulness‐based processes but do not strictly follow ACT or MBI protocols. In total, 60 RCTs were included in the systematic review, of which 51 were eligible for meta‐analysis. The study selection process is illustrated in Figure 1. References for the studies included in the review are presented in Data S1.

### 3.2 Study and Sample Characteristics

The characteristics of the 60 RCTs included in the systematic review are summarized in Table 1. The studies were published between 2008 and 2025 and were conducted across 18 countries: Sweden (*k* = 13), Spain (*k* = 10), the United States (*k* = 10), the United Kingdom (*k* = 7), Norway (*k* = 3), Denmark (*k* = 2), Germany (*k* = 2), Ireland (*k* = 2), Canada (*k* = 2), and one study each from France, India, Iran, Israel, Italy, Japan, Netherlands, New Zealand and China. The RCTs evaluated a range of chronic pain conditions, with unspecified chronic pain (*k* = 31), chronic low back pain (*k* = 10) and fibromyalgia (*k* = 9) being the most common, followed by osteoarthritis (*k* = 3), chronic musculoskeletal pain (*k* = 2), vestibulodynia (*k* = 2), chronic widespread pain (*k* = 1), endometriosis (*k* = 1) and vulvodynia (*k* = 1).

**TABLE 1:** Study characteristics of the 60 RCTs included in the systematic review.

First author (year)	Country	Pain diagnosis	*N* total	Intervention					Control				
				Group (*n*)	Age *M* (SD or range)	Female %	Years of diagnosis, *M* (SD)	Pain intensity, *M* (SD)	Group (*n*)	Age, *M* (SD or range)	Female *%*	Years of diagnosis, *M* (SD)	Pain intensity, *M* (SD)
Morone et al. (2008)	United States	Chronic low back pain	37	MBI (19)	74.1 (6.1)	53	—	17.3 (9.2)^d^	Waitlist (18)	75.6 (5)	61	—	16.2 (7.8)^d^
Wicksell et al. (2008)	Sweden	Chronic pain	21	ACT (11)	48.2 (7.8)	82	7 (3.5)	5.3 (1)^e^	Waitlist (10)	55.1 (11.2)	70	6.8 (3.5)	6.3 (1.5)^e^
Johnston et al. (2010)^a^	New Zealand	Chronic pain	24	ACT (12)	43 (20 to 84)	75	—	21.2 (10.9)^d^	Waitlist (12)	43 (20 to 84)	50	—	20.5 (11.9)^d^
Thorsell et al. (2011)^a^ ^,b^	Sweden	Chronic pain	90	ACT (45)	46 (12.3)	64	—	7.9 (0.2)^f^	Relaxation(45)	46 (12.3)	64	—	8.3 (0.3)^f^
Wetherell et al. (2011)^a^ ^,^ ^b^ ^,^ ^c^	United States	Chronic pain	114	ACT (57)	54.9 (12.5)	51	15 (13.2)	5.9 (1.5)^g^	CCBT (57)	54.9 (12.5)	51	15 (13.2)	5.2 (1.6)^g^
Buhrman et al. (2013)	Sweden	Chronic pain	76	ACT (38)	48.8 (9.5)	55	13.1 (10.6)	4.5 (0.8)^h^	Discussion forum (38)	49.3 (11.3)	63	17.4 (12.4)	4.3 (1)^h^
Kristjánsdóttir et al. (2013a, 2013b)	Norway	Chronic widespread pain	135	ACT (69)	44.6 (11.1)	100	13.1 (8.8)	6.7 (1.7)^e^	Self‐help website (66)	43.8 (11.2)	100	15.5 (12.1)	5.8 (2.2)^e^
McCracken et al. (2013)^a^	United Kingdom	Chronic pain	73	ACT (37)	58 (12.8)	69	17.9 (10.7)	6.5 (1.9)^e^	TAU (36)	58 (12.8)	69	15 (8.1)	6.4 (1.9)^e^
Wicksell et al. (2013)^a^	Sweden	Fibromyalgia	40	ACT (23)	45.1 (6.6)	100	15 (13.5)	4.2 (1)^e^	Waitlist (17)	45.1 (6.6)	100	15 (13.5)	4.3 (1.1)^e^
Luciano et al. (2014)^b^	Spain	Fibromyalgia	155	ACT (51)	48.9 (5.9)	96	14.1 (8.9)	6.5 (1.8)^e^	Waitlist (53)	48.3 (5.7)	94	12.9 (8.7)	6.4 (1.9)^e^
									RPT (51)	47.8 (5.9)	98	11.4 (8)	6.3 (1.6)^e^
Alonso‐Fernández et al. (2015)^a^	Spain	Chronic musculoskeletal pain	64	ACT + SOC (30)	83.1 (6.8)	78	21.3 (20.8)	5.3 (2.3)^g^	SOC (34)	83.1 (6.8)	78	25.3 (20.4)	5.3 (2.1)^g^
Buhrman et al. (2015)^c^	Sweden	Chronic pain		CCBT (52)	54.1 (11.8)	86	—	3.8 (1.1)^h^	Waitlist (24)	46.8 (12.9)	83	—	3.8 (1.1)^h^
Dowd et al. (2015)^a^	Ireland	Chronic pain	124	MBCT (62)	44.5 (12.3)	90	10.3 (9.8)	5.6 (0.2)^g^	PE (62)	44.5 (12.3)	90	11.5 (12.4)	5.9 (0.2)^g^
Kemani et al. (2015)	Sweden	Chronic pain	60	ACT (30)	38.7 (11.1)	80	8.8 (6.2)	4.3 (0.8)^e^	Relaxation(30)	42 (11.6)	67	10.9 (8.5)	4.4 (1)^e^
la Cour and Petersen (2015)	Denmark	Chronic pain	109	MBSR (54)	46.5 (12.4)	83	7.8 (5.5)	19 (6.6)^g^	Waitlist (55)	48.8 (12.2)	87	11.8 (11.1)	19.2 (5.2)^g^
Pincus et al. (2015)^c^	United Kingdom	Chronic low back pain	105	CCBT (45)	43.7 (16.3)	60	3.5 (3.2)	14.9 (8.8)^g^	PT (60)	45.4 (15.8)	61	3.7 (3)	15 (8.3)^g^
Trompetter et al. (2015)^b^	Netherlands	Chronic pain	161	ACT (238)	52.9 (13.3)	77	—	6.3 (1.6)^e^	Waitlist (77)	53.2 (12)	75	—	6.2 (1.6)^e^
									Expressive writing (79)	52.3 (11.8)	76	—	6.1 (1.6)^e^
Henriksson et al. (2016)	Sweden	Chronic pain	107	MBI (55)	52.4 (10.8)	93	15.1 (12.5)	6.2 (1.3)^e^	Discussion forum (52)	50.1 (9.9)	94	13.5 (9.3)	6.4 (1.8)^e^
Turner et al. (2016)	United States	Chronic low back pain	341	MBSR (116)	50 (11.9)	61	—	6 (1.5)e	TAU (113)	48.9 (12.5)	77	—	6 (1.6)e
				CCBT (112)	41.9 (12.6)	59	—	6 (1.5)e					
Zgierska et al. (2016)^a^	United States	Chronic low back pain	35	MBI (21)	51.8 (9.7)	—	—	6.3 (1.2)^g^	Enhanced TAU (14)	51.8 (9.7)	—	—	4.9 (1.1)^g^
Clarke et al. (2017)	United Kingdom	Osteoarthritis	31	ACT (16)	66 (7.3)	75	—	7.7 (2.2)e	TAU (15)	67 (10.7)	67	—	7.3 (0.7)e
Herbert et al. (2017)^a^, ^b^, ^c^	United States	Chronic pain	128	In‐person ACT (65)	52 (13.3)	18	—	6 (0.3)^g^	Online ACT (63)	52 (13.3)	18	—	6.1 (0.2)^g^
Lin et al. (2017)	Germany	Chronic pain	302	Guided ACT (100)	51.7 (12.3)	86	9.7 (11.4)	5.4 (1.5)^e^	Waitlist (101)	50.3 (12.5)	85	7.9 (8.2)	5.1 (1.6)e
				Unguided ACT (101)	53.1 (14.2)	81	11 (10.4)	5.2 (1.5)^e^					
Montero‐Marín et al. (2018)	Spain	Fibromyalgia	42	MBI (23)	50.8 (8.7)	100	—	—	Relaxation (19)	52.2 (5.9)	100	—	—
Scott et al. (2018)	United Kingdom	Chronic pain	63	ACT (31)	47.3 (14)	68	15.9 (11.4)	7.5 (1.3)e	RPT (32)	43.8 (13.9)	59	10.4 (7.3)	7.3 (1.2)e
Simister et al. (2018)^a^	United Kingdom	Fibromyalgia	67	ACT (33)	39.7 (9.4)	95	10.2 (7.8)	26.1 (8.4)^d^	TAU (34)	39.7 (9.4)	95	10.2 (7.8)	25.8 (8.4)^d^
Pérez‐Aranda et al. (2019)	Spain	Fibromyalgia	225	MBSR (75)	52.9 (7.9)	97	13.9 (8.9)	—	TAU (75)	52.7 (8.5)	99	13.1 (9.7)	—
									PE (75)	54.2 (7.4)	99	11.2 (7)	
Veillette et al. (2019)	Canada	Chronic pain	130	ACT (64)	51.9 (14.2)	84	—	5.7 (1.6)e	Waitlist (66)	50.2 (11)	79	—	5.9 (1.4)e
Dindo et al. (2020)	United States	Chronic pain	32	ACT (20)	37.7 (6.3)	0	—	5.5 (0.4)^g^	TAU (12)	34.7 (5.8)	0	—	4.5 (0.5)^g^
Godfrey et al. (2020)^c^	United Kingdom	Chronic low back pain	248	ACT + PT (124)	48.4 (14.6)	61	—	6.1 (2.1)^e^	PT (124)	47.5 (14)	57	—	6.1 (1.9)^e^
Rickardsson et al. (2020)	Sweden	Chronic pain	113	ACT (57)	48.4 (13.1)	72	18.9 (12.7)	5.2 (1.5)^e^	Waitlist (56)	50.6 (11.1)	79	17.3 (13.6)	5.8 (1.5)^e^
Roslyakova et al. (2020)	France	Chronic pain	94	ACT + PRT (35)	49.2 (1.6)	76	12.2 (2.4)	6.9 (1.8)^e^	PRT (59)	46.4 (1.8)	84	7.9 (0.9)	6.9 (1.3)^e^
Scott et al. (2020)	United Kingdom	Chronic pain	38	ACT (25)	55.8 (5.6)	20	12.3 (7.9)	6.8 (2.3)^g^	Waitlist (13)	56 (6.2)	31	9.9 (8.8)	5.9 (1.7)^g^
Taheri et al. (2020)	Iran	Chronic pain	50	ACT (25)	58.6 (9.3)	75	—	—	TAU (25)	56 (9.7)	60	—	—
Bendelin et al. (2021)	Sweden	Chronic pain	103	ACT + MPRP (49)	36.4 (9.7)	88	8.4 (7.7)	6.9 (1.8)^e^	MPRP (54)	35.9 (9.8)	83	5.9 (6)	7.2 (1.5)^e^
Buhler et al. (2021)^a^	Canada	Chronic pain	40	ACT (23)	51.2 (9.3)	31	17.5 (11.3)	—	Waitlist (17)	51.2 (9.3)	31	17.5 (11.3)	—
Torrijos‐Zarcero et al. (2021)^b^, ^c^	Spain	Chronic pain	123	MBI (62)	48.3 (10.2)	90	—	7.5 (1.5)^e^	CBT (61)	49.3 (11.4)	85	—	7.5 (1.4)^e^
Braun et al. (2022)	Germany	Chronic pain	81	ACT (43)	57.2 (9.5)	70	—	4.4 (1.8)^e^	TAU (38)	56.7 (7.7)	71	—	4.3 (1.6)^e^
Casey et al. (2022)	Ireland	Chronic pain	175	ACT + EX (87)	48.4 (10.4)	69	8.8 (7.5)	6.2 (1.6)^g^	PT (88)	47.7 (11.7)	73	10.1 (8.3)	6.5 (1.6)^g^
Diez et al. (2022)^a^	Spain	Chronic low back pain	63	MBSR (31)	53 (−)	66	—	—	TAU (32)	53 (−)	66	—	—
Hess Engström et al. (2022)	Sweden	Vulvodynia	88	ACT (49)	24.2 (5.2)	100	5.2 (4.7)	6.8 (2.4)^e^	Waitlist (39)	24.7 (3.3)	100	4.6 (3.5)	6.9 (1.8)^e^
Gasslander et al. (2022)^c^	Sweden	Chronic musculoskeletal pain	187	CCBT (95)	45.6 (11.1)	74	15.4 (11.1)	4 (0.8)^h^	Waitlist (92)	46.2 (11.2)	73	14.3 (9.8)	3.9 (1.1)^h^
Kanzler et al. (2022)	United States	Chronic pain	26	ACT (13)	54 (−)	63	9.7 (7.4)	6.5 (1.9)^e^	Enhanced TAU (13)	50 (−)	46	14 (13.1)	7.1 (1.6)^e^
Nagasawa et al. (2022)	Japan	Osteoarthritis	30	ACT (15)	73.3 (7)	93	5.3 (5.4)	4.1 (1.8)^e^	PT (15)	75.1 (6.9)	87	2.3 (2.4)	4.1 (1.7)^e^
Pérez‐Fernández et al. (2022)	Spain	Chronic pain	90	MBI (50)	52.5 (9.1)	90	—	2.7 (0.6)^i^	Waitlist (40)	51.5 (8.9)	88	—	2.6 (0.7)^i^
Plumb et al. (2022)^a^	United States	Osteoarthritis	39	ACT (19)	71.8 (5.2)	85	—	13.7 (3.8)^j^	Enhanced TAU (20)	71.8 (5.2)	85	—	14.9 (4.2)^j^
Bostrøm et al. (2023)	Norway	Chronic pain	259	CCBT (125)	50 (−)	82	—	—	TAU (134)	48 (−)	80	—	—
Hansen et al. (2023)	Denmark	Endometriosis	39	MBSR + ACT (19)	28.9 (7.8)	100	14 (8.3)	6.1 (2.1)^e^	NPSI (19)	33.8 (7.7)	100	18.2 (6)	5.5 (1.9)
									Waitlist (16)	32.8 (9)	100	12.9 (7.5)	6 (1.5)
Maathz et al. (2023)	Sweden	Vestibulodynia	37	ACT (16)	25.1 (2.9)	100	18.5 (3.2)	—	Waitlist (21)	28.2 (6.1)	100	21.5 (5.5)	—
Sanabria‐Mazo, Colomer‐Carbonell, Borràs, et al. (2023), Sanabria‐Mazo, Colomer‐Carbonell, Fernández‐Vázquez, et al. (2023)	Spain	Chronic low back pain	234	ACT (78)	54.9 (8.3)	69	10.9 (7.9)	6.9 (1.7)^e^	TAU (78)	53.8 (10)	65	11.2 (8)	6.9 (1.7)^e^
				BATD (78)	54.9 (10.2)	68	11.1 (8.7)	6.5 (1.7)^e^					
Varallo et al. (2023)	Italy	Fibromyalgia	180	ACT (90)	45.2 (6.4)	100	—	4.9 (1.9)^e^	Enhanced TAU (90)	44.7 (6.8)	100	—	4.4 (1.2)^e^
Buhrman et al. (2024)	Sweden	Vestibulodynia	88	ACT (46)	27 (5.2)	100	—	1.6 (1.3)^h^	Waitlist (42)	25 (4.6)	100	—	1.8 (1.5)^h^
Gendreau et al. (2024)	United States	Fibromyalgia	275	ACT (140)	49 (13.7)	92	9.3 (11.1)	—	PE (135)	49 (11.9)	94	8.7 (8.9)	—
Solberg Nes et al. (2024)^c^	Norway	Chronic pain	159	CCBT (125)	50 (−)	82	—	5.1 (1.7)^g^	TAU (134)	48 (−)	80	—	5.4 (1.5)^g^
Pal et al. (2024)	India	Chronic low back pain	195	MBSR (98)	48.7 (5.9)	30	—	7 (1)^e^	PRT (97)	49.3 (5.9)	26	—	7.2 (0.8)^e^
Ramos et al. (2024)	Spain	Fibromyalgia	57	ACT (32)	51.9 (8.3)	100	14.5 (8.3)	12.5 (13.4)^k^	Waitlist (25)	52.1 (9.5)	100	17.5 (9.5)	9.7 (12.1)^k^
Reilly et al. (2024)	United States	Chronic pain	113	ACT (57)	50.6 (16.6)	25	—	7.2 (1.7)^e^	Waitlist (56)	56.5 (16.6)	14	—	7 (1.7)^e^
Robles et al. (2024)	Spain	Chronic low back pain	32	MBSR (19)	52.4 (9.6)	73	—	4.3 (0.7)^e^	Waitlist (13)	59 (10.1)	67	—	4.4 (0.9)^e^
Taub et al. (2024)	Israel	Fibromyalgia	81	MBSR (41)	50.5 (12.4)	88	6.5 (5.2)	—	Waitlist (40)	48.2 (14)	14	8.4 (6.9)	—
Liu et al. (2025)	China	Chronic low back pain	40	ACT + EX (20)	72.8 (6.2)	95	—	5.2 (1.8)^e^	EX (20)	73.6 (6.2)	85	—	5.1 (1.9)^e^

*Note:* Studies are ordered chronologically by year of publication (oldest to most recent); within the same year, studies are listed alphabetically by first author.

Abbreviations: ACT, acceptance and commitment therapy; CCBT, contextual cognitive‐behavioural therapy; EX, exercise; MBI, mindfulness‐based intervention; MBSR, mindfulness‐based stress reduction; MPRP, multimodal pain rehabilitation program; NPSI, non‐specific psychological intervention (without mindfulness/ACT components); PE, psychoeducation; PRT, psychoeducation relaxation therapy; PT, physical therapy; RPT, recommended pharmacological treatment; SOC, selective optimization with compensation.

^a^ Only mean age, pain intensity, years of diagnosis (with standard deviation) and/or percentage of females were reported for the total sample.

^b^ CCBT or online ACT were included as comparators.

^c^ This study was included in the systematic review but was not eligible for the meta‐analyses.

^d^ Assessed with the McGill Pain Questionnaire (MPQ).

^e^ Assessed with the Visual Analogue Scale (VAS) or Numeric Rating Scale (NRS).

^f^ Assessed with the Örebro Musculoskeletal Pain Questionnaire (ÖMPQ).

^g^ Assessed with the Brief Pain Inventory (BPI).

^h^ Assessed with the Multidimensional Pain Inventory (MPI).

^i^ Assessed with the Lattinen Index (LI).

^j^ Assessed with the Arthritis Impact Measurement Scale (AIMS).

^k^ Assessed with the SF‐36 Health Survey Questionnaire.

In total, the RCTs enrolled 6692 participants, with 3491 assigned to intervention groups and 3201 to control groups. Sample sizes ranged from 21 to 341 participants, with intervention groups ranging from 11 to 238 and control groups from 10 to 135 participants. Mean participant ages ranged from 24 to 83 years in intervention groups and from 25 to 83 years in control groups. Most RCTs included samples with a higher proportion of females. Nine trials recruited only female participants (Buhrman et al. 2024; Hansen et al. 2023; Hess Engström et al. 2022; Kristjánsdóttir et al. 2013b; Maathz et al. 2023; Montero‐Marín et al. 2018; Ramos et al. 2024; Varallo et al. 2023; Wicksell et al. 2013), and one recruited only male participants (Dindo et al. 2020). The mean duration of pain diagnosis ranged from 2 to 25 years in both intervention and control groups, and baseline pain intensity scores, mostly assessed with 0–10 rating scales such as the Visual Analog Scale (VAS), the Numeric Rating Scale (NRS) or the Brief Pain Inventory (BPI), ranged from 4 to 8 across groups.

### 3.3 Intervention and Control Characteristics

Interventions were based on ACT (*k* = 40) (Alonso‐Fernández et al. 2015; Bendelin et al. 2021; Braun et al. 2022; Buhler et al. 2021; Buhrman et al. 2013, 2024; Casey et al. 2022; Clarke et al. 2017; Dindo et al. 2020; Gendreau et al. 2024; Godfrey et al. 2020; Herbert et al. 2017; Hess Engström et al. 2022; Johnston et al. 2010; Kanzler et al. 2022; Kemani et al. 2015; Kristjánsdóttir et al. 2013b; Lin et al. 2017; Liu et al. 2025; Luciano et al. 2014; Maathz et al. 2023; McCracken et al. 2013; Nagasawa et al. 2022; Plumb et al. 2022; Ramos et al. 2024; Reilly et al. 2024; Rickardsson et al. 2020; Roslyakova et al. 2020; Sanabria‐Mazo, Colomer‐Carbonell, Borràs, et al. 2023; Scott et al. 2018, 2020; Simister et al. 2018; Taheri et al. 2020; Thorsell et al. 2011; Trompetter et al. 2015; Varallo et al. 2023; Veillette et al. 2019; Wetherell et al. 2011; Wicksell et al. 2008, 2013), MBI (*k* = 15) (Diez et al. 2022; Dowd et al. 2015; Hansen et al. 2023; Henriksson et al. 2016; la Cour and Petersen 2015; Montero‐Marín et al. 2018; Morone et al. 2008; Pal et al. 2024; Pérez‐Aranda et al. 2019; Pérez‐Fernández et al. 2022; Robles et al. 2024; Taub et al. 2024; Torrijos‐Zarcero et al. 2021; Turner et al. 2016; Zgierska et al. 2016), and CCBT (*k* = 6) (Bostrøm et al. 2023; Buhrman et al. 2015; Gasslander et al. 2022; Pincus et al. 2015; Solberg Nes et al. 2024). Counts exceed 60 because some trials included multiple comparator arms and/or multiple providers. The study by Hansen et al. (2023), which combined MBSR with ACT, was classified as MBI because MBSR formed the core protocol.

Most interventions were delivered as stand‐alone programs (*k* = 49). Combined programs (*k* = 12) included combinations such as ACT with physical therapy (Godfrey et al. 2020), ACT with exercise (Casey et al. 2022; Liu et al. 2025), ACT with psychoeducation relaxation therapy (Roslyakova et al. 2020) and ACT with selective optimization plus compensation (SOC) (Alonso‐Fernández et al. 2015), as well as CCBT protocols integrating acceptance‐ and/or mindfulness strategies with additional therapeutic ingredients (Bostrøm et al. 2023; Buhrman et al. 2015; Gasslander et al. 2022; Pincus et al. 2015; Solberg Nes et al. 2024; Turner et al. 2016).

Intervention duration ranged from 1 day to 48 weeks (*M* = 8.5, SD = 5.5), and the number of sessions/modules ranged from 1 to 15 (*M* = 7.7, SD = 2.6). Session length ranged from 10 to 300 min (*M* = 100.6), with longer formats typically delivered face‐to‐face and shorter modules delivered via web‐ or app‐based platforms. Delivery modes comprised face‐to‐face (*k* = 32), online (*k* = 24), blended (*k* = 4) and phone‐based (*k* = 1). Treatment formats included individual (*k* = 31), group (*k* = 29) or combined individual and group delivery (*k* = 1). Regarding involvement, most were therapist‐delivered (*k* = 49), followed by self‐guided (*k* = 9) and mixed therapist‐delivered and self‐guided approaches (*k* = 3). Providers were primarily psychologists (*k* = 46), with additional contributions from MBSR instructors (*k* = 4), researchers (*k* = 4), physiotherapists (*k* = 2) and one physician (*k* = 1). In some interventions, no provider was involved as they were entirely self‐guided (*k* = 4).

Control groups included inactive comparators (*k* = 35), such as waitlist (*k* = 23) and treatment as usual (TAU; *k* = 12) and active comparators (*k* = 29), such as relaxation training (*k* = 5), enhanced TAU (*k* = 4), physical therapy (*k* = 4), psychoeducation (*k* = 3), discussion forums (*k* = 2), recommended pharmacological treatment (RPT; *k* = 2), CCBT (*k* = 2), expressive writing (*k* = 1), exercise (*k* = 1), non‐specific psychological interventions (*k* = 1), online ACT (*k* = 1), multimodal pain rehabilitation program (*k* = 1), SOC (*k* = 1) and self‐help website (*k* = 1). Assessments were conducted at post‐treatment only (*k* = 16), one follow‐up (*k* = 38) or two follow‐ups (*k* = 6), with follow‐up periods ranging from 4 to 60 weeks after baseline. Dropout rates in intervention groups ranged from 0% to 70%, and in control groups from 0% to 71%.

Psychological flexibility and its dimensions were assessed with a range of validated instruments. The global construct of psychological flexibility (*k* = 18) was evaluated using the PIPS (*k* = 12) and the AAQ‐II (*k* = 6). At the dimension level, acceptance was measured with the Chronic Pain Acceptance Questionnaire (CPAQ; *k* = 47); cognitive defusion with the Cognitive Fusion Questionnaire (CFQ; *k* = 3); values with the Chronic Pain Values Inventory (CPVI; *k* = 3) and Valuing Questionnaire (VQ; *k* = 1); and committed action with the Committed Action Questionnaire (CAQ; *k* = 4) and the Engaged Living Scale (ELS; *k* = 2). Regarding outcome role, psychological flexibility and its dimensions were specified as a primary outcome (*k* = 4), secondary outcome (*k* = 24) or process variable (*k* = 20); in some studies, this was not specified (*k* = 12).

A detailed description of intervention and control characteristics for all included RCTs is provided in Table 2. In addition, the results of each included study are available in Data S2.

**TABLE 2:** Characteristics of intervention and control arms of the included RCTs (*k* = 60).

First author (year)	Therapy (structure)	Duration (sessions/modules)	Session/module duration	Delivery (format)	Provider (involvement)	Control (type)	Assessments (weeks from baseline)	Dropout intervention (%)	Dropout control (%)	Outcomes (instruments)
Morone et al. (2008)	MBI (stand‐alone)	8 weeks (8 sessions)	90 min	Face‐to‐face (group)	MBSR instructor (therapist‐delivered)	Waitlist (inactive)	Pre (0) Post (8)	Post (32)	Post (6)	Pain acceptance (CPAQ)
Wicksell et al. (2008)	ACT (stand‐alone)	8 weeks (10 sessions)	60 min	Face‐to‐face (individual)	Psychologist (therapist‐delivered)	Waitlist (inactive)	Pre (0) Post (8) FW (16)	Post (0) FW (16)	Post (10) FW (9)	Psychological inflexibility (PIPS)
Johnston et al. (2010)	ACT (stand‐alone)	6 weeks (6 sessions)	—	Blended (individual)	Researcher (self‐guided)	Waitlist (inactive)	Pre (0) Post (6)	Post (50)	Post (33)	Pain acceptance (CPAQ) Values (CPVI)
Thorsell et al. (2011)	ACT (stand‐alone)	7 weeks (7 sessions)	90 min	Blended (individual)	Psychologist (therapist‐delivered)	Relaxation (active)	Pre (0) Post (7) FW 1 (31) FW 2 (55)	Post (22) FW 1 (33) FW 2 (44)	Post (13) FW 1 (24) FW 2 (38)	Pain acceptance (CPAQ)
Wetherell et al. (2011)^a^	ACT (stand‐alone)	8 weeks (8 sessions)	90 min	Face‐to‐face (group)	Psychologist (therapist‐delivered)	CCBT (active)	Pre (0) Post (8) FW (32)	Post (−) FW (−)	Post (−) FW (−)	Pain acceptance (CPAQ)
Buhrman et al. (2013)	ACT (stand‐alone)	7 weeks (7 sessions)	—	Online—asynchronous (individual)	Psychologist (therapist‐delivered)	Discussion forum (active)	Pre (0) Post (7) FW (24)	Post (17) FW (17)	Post (16) FW (−)	Pain acceptance (CPAQ)
Kristjánsdóttir et al. (2013a, 2013b)	ACT (stand‐alone)	4 weeks (1 session)	60 min	Online—asynchronous (individual)	Psychologist (therapist‐delivered)	Self‐help website (active)	Pre (0) Post (4) FW 1 (28) FW 2 (56)	Post (33) FW 1 (47) FW 2 (44)	Post (47) FW 1 (43) FW 2 (36)	Pain acceptance (CPAQ)
McCracken et al. (2013)	ACT (stand‐alone)	2 weeks (4 sessions)	240 min	Face‐to‐face (group)	Psychologist (therapist‐delivered)	TAU (inactive)	Pre (0) Post (4) FW (12)	Post (16) FW (24)	Post (25) FW (22)	Psychological inflexibility (AAQ‐II) Pain acceptance (CPAQ)
Wicksell et al. (2013)	ACT (stand‐alone)	12 weeks (12 sessions)	90 min	Face‐to‐face (group)	Psychologist/medical staff (therapist‐delivered)	Waitlist (inactive)	Pre (0) Post (12) FW (24)	Post (13) FW (17)	Post (6) FW (18)	Psychological inflexibility (PIPS)
Luciano et al. (2014)	ACT (stand‐alone)	8 weeks (8 sessions)	150 min	Face‐to‐face (group)	Psychologist (therapist‐delivered)	Waitlist (inactive)	Pre (0) Post (8) FW (24)	Post (10) FW (12)	Post (6) FW (11)	Pain acceptance (CPAQ)
						RPT (active)				
Alonso‐Fernández et al. (2015)	ACT + SOC (combined)	9 weeks (9 sessions)	120 min	Face‐to‐face (group)	Psychologist (therapist‐delivered)	SOC (active)	Pre (0) Post (9)	Post (10)	Post (24)	Pain acceptance (CPAQ)
Buhrman et al. (2015)^a^	CCBT (combined)	8 weeks (8 sessions)	—	Online – asynchronous (individual, tailored)	Psychologist (therapist‐delivered)	Waitlist (inactive)	Pre (0) Post (6)	Post (18)	Post (17)	Pain acceptance (CPAQ)
Dowd et al. (2015)	MBCT (stand‐alone)	6 weeks (12 sessions)	20 min	Online—asynchronous (individual)	None (self‐guided)	PE (active)	Pre (0) Post (6) FW (24)	Post (55) FW (63)	Post (40) FW (57)	Pain acceptance (CPAQ)
Kemani et al. (2015)	ACT (stand‐alone)	12 weeks (12 sessions)	90 min	Face‐to‐face (group)	Psychologist/medical staff (therapist‐delivered)	Relaxation (active)	Pre (0) Post (12) FW 1 (36) FW 2 (60)	Post (20) FW 1 (20) FW 2 (37)	Post (37) FW 1 (40) FW 2 (40)	Pain acceptance (CPAQ)
la Cour and Petersen (2015)	MBSR (stand‐alone)	9 weeks (9 sessions)	180 min	Face‐to‐face (group)	MBSR instructor (therapist‐delivered)	Waitlist (inactive)	Pre (0) Post (9) FW (33)	Post (20) FW (26)	Post (15) FW (40)	Pain acceptance (CPAQ)
Pincus et al. (2015)^a^	CCBT (combined)	8 weeks (8 sessions)	50 min	Face‐to‐face (individual)	Psychologist (therapist‐delivered)	PT (active)	Pre (0) Post (8) FW (24)	Post (27) FW (42)	Post (27) FW (36)	Psychological inflexibility (AAQ‐II) Pain acceptance (CPAQ)
Trompetter et al. (2015)	ACT (stand‐alone)	12 weeks (9 modules)	10–15 min	Online – asynchronous (individual)	Psychologist (therapist‐delivered and self‐guided)	Waitlist (inactive)	Pre (0) Post (12) FW (36)	Post (28) FW (35)	Post (36) FW (37)	Psychological inflexibility (PIPS) Committed action (ELS)
						Expressive writing (active)				
Henriksson et al. (2016)	MBI (stand‐alone)	8 weeks (8 sessions)	10 min	Online—asynchronous (individual)	None (self‐guided)	Discussion forum (active)	Pre (0) Post (8)	Post (35)	Post (21)	Pain acceptance (CPAQ)
Turner et al. (2016)	MBSR (stand‐alone)	8 weeks (8 sessions)	120 min	Face‐to‐face (group)	MBSR instructor (therapist‐delivered)	TAU (inactive)	Pre (0) Post (8) FW 1 (26) FW 2 (52)	Post (−) FW 1 (−) FW 2 (−)	Post (−) FW 1 (−) FW 2 (−)	Pain acceptance (CPAQ)
	CCBT (combined)				Psychologist (therapist‐delivered)					
Zgierska et al. (2016)	MBI (stand‐alone)	8 weeks (8 sessions)	120 min	Face‐to‐face (group)	Psychologist (therapist‐delivered)	Enhanced TAU (active)	Pre (0) Post (8) FW (26)	Post (0) FW (0)	Post (0) FW (0)	Pain acceptance (CPAQ)
Clarke et al. (2017)	ACT (stand‐alone)	6 weeks (6 sessions)	90 min	Face‐to‐face (group)	Psychologist (therapist‐delivered)	TAU (inactive)	Pre (0) Post (8) FW (16)	Post (6) FW (19)	Post (27) FW (47)	Pain acceptance (CPAQ)
Herbert et al. (2017)^a^	In‐person ACT (stand‐alone)	8 weeks (8 sessions)	60 min	Online – synchronous (individual)	Psychologist (therapist‐delivered)	Online ACT (active)	Pre (0) Post (8) FW (32)	Post (29) FW (37)	Post (14) FW (19)	Pain acceptance (CPAQ)
Lin et al. (2017)	Guided ACT (stand‐alone)	8 weeks (8 sessions)	60 min	Online – asynchronous (individual)	Psychologist (therapist‐delivered)	Waitlist (inactive)	Pre (0) Post (9) FW (24)	Post (31) FW (46)	Post (11) FW (26)	Pain acceptance (CPAQ)
	Unguided ACT (stand‐alone)				None (self‐guided)					
Montero‐Marín et al. (2018)	MBI (stand‐alone)	8 weeks (11 sessions)	120 min	Face‐to‐face (group)	Psychologist (therapist‐delivered)	Relaxation (active)	Pre (0) Post (8) FW (12)	Post (13) FW (13)	Post (16) FW (13)	Psychological inflexibility (AAQ‐II)
Scott et al. (2018)	ACT (stand‐alone)	12 weeks (8 sessions)	20 min	Online – asynchronous (individual)	Psychologist (therapist‐delivered)	RPT (active)	Pre (0) Post (12) FW (36)	Post (26) FW (26)	Post (22) FW (19)	Pain acceptance (CPAQ) Commited action (CAQ)
Simister et al. (2018)	ACT (stand‐alone)	8 weeks (7 sessions)	—	Online – asynchronous (individual)	Psychologist (therapist‐delivered)	TAU (inactive)	Pre (0) Post (8) FW (20)	Post (10) FW (17)	Post (0) FW (19)	Pain acceptance (CPAQ) Cognitive fusion (CFQ) Values (VLQ)
Pérez‐Aranda et al. (2019)	MBSR (stand‐alone)	8 weeks (8 sessions)	120 min	Face‐to‐face (group)	Psychologist (therapist‐delivered)	TAU (inactive)	Pre (0) Post (6) FW (48)	Post (23) FW (35)	Post (24) FW (33)	Psychological inflexibility (PIPS)
						PE (active)				
Veillette et al. (2019)	ACT (stand‐alone)	8 weeks (8 sessions)	60–240 min	Online – asynchronous (individual)	Psychologist (self‐guided)	Waitlist (inactive)	Pre (0) Post (8) FW (21)	Post (20) FW (47)	Post (20) FW (−)	Psychological inflexibility (PIPS) Pain acceptance (CPAQ)
Dindo et al. (2020)	ACT (stand‐alone)	1 day (1 session)	300 min	Face‐to‐face (group)	Psychologist (therapist‐delivered)	TAU (inactive)	Pre (0) Post (12)	Post (26)	Post (17)	Psychological inflexibility (AAQ‐II)
Godfrey et al. (2020)^a^	ACT + PT (combined)	3 weeks (3 sessions)	60 min	Blended (individual)	Physiotherapist (therapist‐delivered)	PT (active)	Pre (0) Post (12) FW (48)	Post (17) FW (22)	Post (17) FW (32)	Pain acceptance (CPAQ) Commited action (CAQ)
Rickardsson et al. (2020)	ACT (stand‐alone)	8 weeks (8 sessions)	—	Online – asynchronous (individual)	Psychologist (self‐guided)	Waitlist (inactive)	Pre (0) Post (8) FW (32)	Post (19) FW (35)	Post (4) FW (4)	Psychological inflexibility (PIPS)
Roslyakova et al. (2020)	ACT + PRT (combined)	15 weeks (15 sessions)	120 min (ACT) + 90 min (PRT)	Face‐to‐face (group)	Psychologist (therapist‐delivered)	PRT (active)	Pre (0) Post (15) FW (27)	Post (11) FW (37)	Post (3) FW (46)	Psychological inflexibility (PIPS) Pain acceptance (CPAQ)
Scott et al. (2020)	ACT (stand‐alone)	8 weeks (12 sessions)	60 min	Online—asynchronous (individual)	Psychologist (therapist‐delivered)	Waitlist (inactive)	Pre (0) Post (8) FW (20)	Post (24) FW (32)	Post (8) FW (23)	Pain acceptance (CPAQ)
Taheri et al. (2020)	ACT (stand‐alone)	8 weeks (8 sessions)	—	Face‐to‐face (group)	Psychologist (therapist‐delivered)	TAU (inactive)	Pre (0) Post (8) FW (20)	Post (20) FW (20)	Post (8) FW (16)	Pain acceptance (CPAQ)
Bendelin et al. (2021)	ACT + MPRP (stand‐alone)	6 weeks (8 sessions)	15 min	Online—asynchronous (individual)	Psychologist (therapist‐delivered)	MPRP (active)	Pre (0) Post (6) FW (48)	Post (20) FW (63)	Post (30) FW (59)	Psychological inflexibility (PIPS) Pain acceptance (CPAQ)
Buhler et al. (2021)	ACT (stand‐alone)	8 weeks (8 sessions)	60 min	Blended (individual + group)	Psychologist (therapist‐delivered and self‐guided)	Waitlist (inactive)	Pre (0) Post (8) FW (20)	Post (39) FW (70)	Post (12) FW (−)	Pain acceptance (CPAQ)
Torrijos‐Zarcero et al. (2021)^a^	MBI (stand‐alone)	8 weeks (8 sessions)	150 min	Face‐to‐face (group)	Psychologist (therapist‐delivered)	CCBT (active)	Pre (0) Post (6)	Post (32)	Post (23)	Pain acceptance (CPAQ)
Braun et al. (2022)	ACT (stand‐alone)	9 weeks (7 sessions)	60 min	Online—asynchronous (individual)	Psychologist (therapist‐delivered)	TAU (inactive)	Pre (0) Post (9) FW 1 (24) FW 2 (48)	Post (39) FW 1 (39) FW 2 (43)	Post (22) FW 1 (33) FW 2 (38)	Pain acceptance (CPAQ) Cognitive fusion (CFQ) Commited action (CAQ)
Casey et al. (2022)	ACT + EX (combined)	8 weeks (8 sessions)	210 min	Face‐to‐face (group)	Psychologist/physiotherapist (therapist‐delivered)	PT (active)	Pre (0) Post (8) FW 1 (12) FW 2 (48)	Post (26) FW 1 (19) FW 2 (49)	Post (19) FW 1 (21) FW 2 (56)	Pain acceptance (CPAQ) Commited action (CAQ)
Diez et al. (2022)	MBSR (stand‐alone)	8 weeks (9 sessions)	210 min	Face‐to‐face (group)	Psychologist/physiotherapist (therapist‐delivered)	TAU (inactive)	Pre (0) Post (8)	Post (5)	Post (0)	Pain acceptance (CPAQ)
Hess Engström et al. (2022)	ACT (stand‐alone)	6 weeks (6 sessions)	—	Online – asynchronous (individual)	Researcher (therapist‐delivered)	Waitlist (inactive)	Pre (0) Post (6) FW (39)	Post (39) FW (48)	Post (28) FW (43)	Pain acceptance (CPAQ)
Gasslander et al. (2022)^a^	CCBT (combined)	10 weeks (6–13 modules)	—	Online—asynchronous (individual, tailored)	Psychologist (therapist‐delivered)	Waitlist (inactive)	Pre (0) Post (12) FW (48)	Post (31) FW (44)	Post (15) FW (71)	Pain acceptance (CPAQ)
Kanzler et al. (2022)	ACT (stand‐alone)	12 weeks (5 sessions)	60 min	Face‐to‐face (individual)	Psychologist (therapist‐delivered)	Enhanced TAU (active)	Pre (0) Post (12) FW (36)	Post (23) FW (23)	Post (0) FW (0)	Pain acceptance (CPAQ) Values (CPVI)
Nagasawa et al. (2022)	ACT (stand‐alone)	8 weeks (8 sessions)	60 min	Face‐to‐face (individual)	Physiotherapist (therapist‐delivered)	PT (active)	Pre (0) Post (8) FW (12)	Post (33) FW (47)	Post (13) FW (53)	Psychological inflexibility (PIPS)
Pérez‐Fernández et al. (2022)	MBI (stand‐alone)	8 weeks (8 sessions)	150 min	Face‐to‐face (group)	Psychologist (therapist‐delivered)	Waitlist (inactive)	Pre (0) Post (8)	Post (20)	Post (3)	Pain acceptance (CPAQ)
Plumb et al. (2022)	ACT (stand‐alone)	6 weeks (2 sessions)	45 min	Phone‐based (individual)	Researcher (therapist‐delivered)	Enhanced TAU (active)	Pre (0) Post (6)	Post (0)	Post (5)	Psychological inflexibility (AAQ‐II)
Bostrøm et al. (2023)^a^	CCBT (combined)	9 weeks (9 sessions)	—	Online – asynchronous (individual)	None (self‐guided)	TAU (inactive)	Pre (0) Post (12)	Post (11)	Post (10)	Pain acceptance (CPAQ)
Hansen et al. (2023)	MBSR + ACT (combined)	10 weeks (10 sessions)	180 min	Face‐to‐face (group)	Psychologist (therapist‐delivered)	NPSI (active)	Pre (0) Post (10)	Post (26)	Post (20)	Pain acceptance (CPAQ)
						Waitlist (inactive)				
Maathz et al. (2023)	ACT (stand‐alone)	6 weeks (6 modules)	—	Online – asynchronous (individual)	Psychologist (therapist‐delivered)	Waitlist (inactive)	Pre (0) Post (7)	Post (19)	Post (0)	Pain acceptance (CPAQ)
Sanabria‐Mazo, Colomer‐Carbonell, Borràs, et al. (2023), Sanabria‐Mazo, Colomer‐Carbonell, Fernández‐Vázquez, et al. (2023)	ACT (stand‐alone)	8 weeks (8 sessions)	90 min	Online – synchronous (group)	Psychologist (therapist‐delivered)	TAU (inactive)	Pre (0) Post (8) FW (48)	Post (40) FW (47)	Post (18) FW (32)	Psychological inflexibility (PIPS) Pain acceptance (CPAQ)
	BATD (stand‐alone)									
Varallo et al. (2023)	ACT (stand‐alone)	3 weeks (3 sessions)	60 min	Face‐to‐face (group)	Psychologist (therapist‐delivered)	Enhanced TAU (active)	Pre (0) Post (3)	Post (4)	Post (7)	Pain acceptance (CPAQ)
Buhrman et al. (2024)	ACT (stand‐alone)	10 weeks (8 modules)	—	Online – asynchronous (individual)	Psychologist (therapist‐delivered)	Waitlist (inactive)	Pre (0) Post (10) FW (48)	Post (18) FW (14)	Post (14) FW (31)	Pain acceptance (CPAQ)
Gendreau et al. (2024)	ACT (stand‐alone)	12 weeks (8 modules)	—	Online – asynchronous (individual)	None (self‐guided)	PE (active)	Pre (0) Post (12)	Post (12)	Post (4)	Psychological inflexibility (PIPS)
Solberg Nes et al. (2024)^a^	CCBT (combined)	48 weeks (9 modules)	—	Online – asynchronous (individual)	Psychologist (self‐guided)	TAU (inactive)	Pre (0) Post (24) FW (48)	Post (26) FW (25)	Post (16) FW (18)	Pain acceptance (CPAQ)
Pal et al. (2024)	MBSR (stand‐alone)	5 weeks (5 sessions)	90 min	Face‐to‐face (group)	Physician (therapist‐delivered)	PRT (active)	Pre (0) Post (5) FW (25)	Post (8) FW (18)	Post (6) FW (16)	Pain acceptance (CPAQ)
Ramos et al. (2024)	ACT (stand‐alone)	5 weeks (5 sessions)	120 min	Face‐to‐face (group)	Psychologist (therapist‐delivered)	Waitlist (inactive)	Pre (0) Post (8) FW (32)	Post (28) FW (28)	Post (0) FW (0)	Cognitive fusion (CFQ)
Reilly et al. (2024)	ACT (stand‐alone)	7 weeks (7 sessions)	—	Online—asynchronous (individual)	Researcher (self‐guided)	Waitlist (inactive)	Pre (0) Post (7) FW (11)	Post (15) FW (20)	Post (14) FW (14)	Pain acceptance (CPAQ) Values (CPVI)
Robles et al. (2024)	MBSR (stand‐alone)	8 weeks (8 sessions)	150 min	Face‐to‐face (group)	MBSR instructor (therapist‐delivered)	Waitlist (inactive)	Pre (0) Post (8)	Post (5)	Post (15)	Pain acceptance (CPAQ)
Taub et al. (2024)	MBSR (stand‐alone)	10 weeks (11 sessions)	120 min	Face‐to‐face (group)	Psychologist (therapist‐delivered)	Waitlist (inactive)	Pre (0) Post (10) FW (34)	Post (16) FW (29)	Post (15) FW (−)	Psychological inflexibility (PIPS)
Liu et al. (2025)	ACT + EX (combined)	8 weeks (8 sessions)	60 min	Face‐to‐face (group)	Psychologist (therapist‐delivered)	EX (active)	Pre (0) Post (8) FW (32)	Post (10) FW (28)	Post (15) FW (15)	Psychological inflexibility (AAQ‐II)

*Note:* Studies are ordered chronologically by year of publication (oldest to most recent); within the same year, studies are listed alphabetically by first author.

Abbreviations: AAQ‐II, Acceptance and Action Questionnaire–II; ACT, acceptance and commitment therapy; BATD, behavioural activation therapy for depression; CAQ, Committed Action Questionnaire; CCBT, contextual cognitive‐behavioural therapy; CFQ, Cognitive Fusion Questionnaire; CPAQ, Chronic Pain Acceptance Questionnaire; CPVI, Chronic Pain Values Inventory; ELS, Engaged Living Scale; EX, exercise; FW, follow‐up; MBI, mindfulness‐based intervention; MBSR, mindfulness‐based stress reduction; MPRP, multimodal pain rehabilitation program; NPSI, non‐specific psychological intervention (without mindfulness/ACT components); PE, psychoeducation; PIPS: Psychological Inflexibility in Pain Scale; PT, physical therapy; PRT, psychoeducation relaxation therapy; RPT, recommended pharmacological treatment; SOC, selective optimization with compensation; VLQ, Valued Living Questionnaire.

^a^ This study was included in the systematic review but was not eligible for the meta‐analyses.

### 3.4 Risk of Bias

**FIGURE 2:**
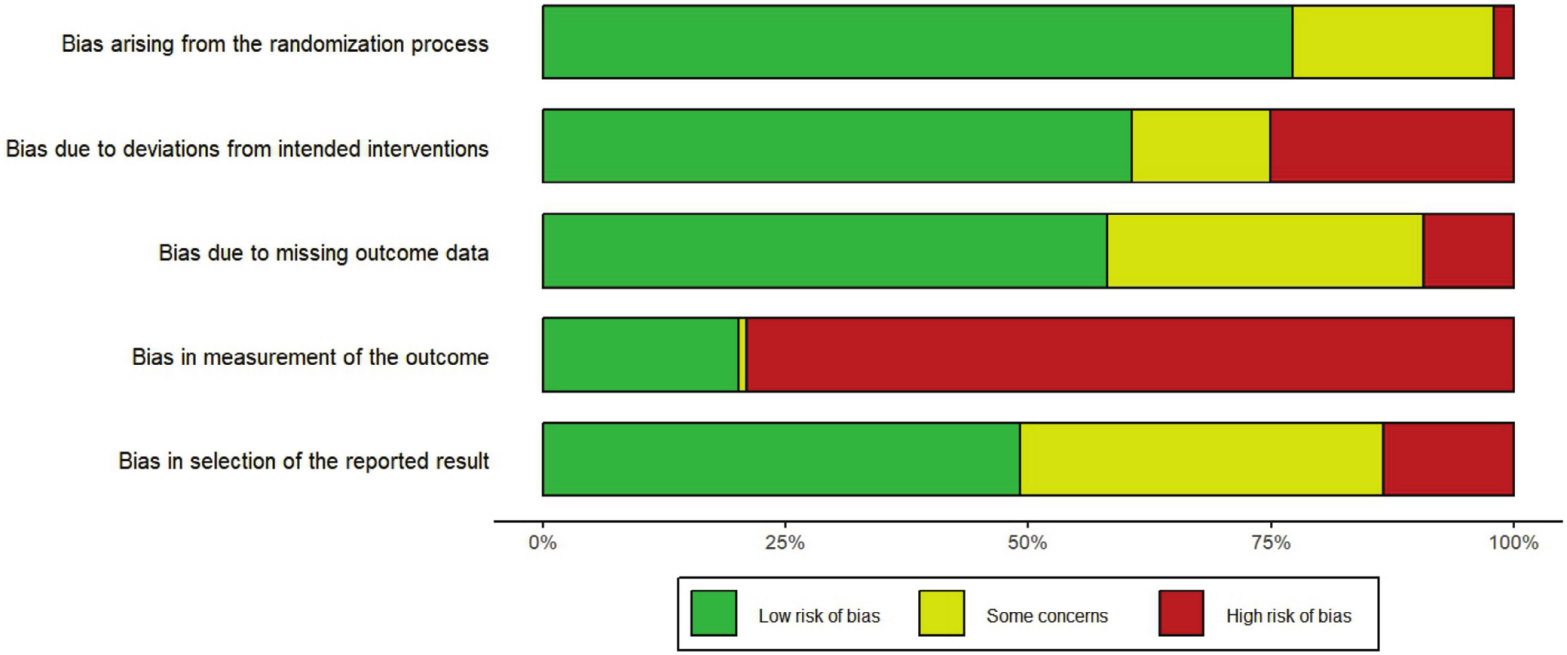
Risk of bias summary across domains.

Figure 2 presents a bar plot summarizing the proportions of studies assigned to each risk‐of‐bias category across domains. Sample size was used as the study weight because not all depicted RCTs were included in the meta‐analyses. Overall, 1 study (2%) was judged low risk, 6 (10%) raised some concerns, and 53 (88%) were high risk. The highest level of concern was observed for bias risk in outcome measurement, with 85% of studies rated as high risk, whereas the remaining domains showed predominantly low or moderate risk. Figure S1 presents the corresponding traffic‐light plot with domain‐level judgements for each study.

### 3.5 Meta‐Analytic Effects

#### 3.5.1 Methodological Considerations

Before conducting the meta‐analyses, specific considerations were necessary to address trials with multiple study arms (*k* = 7). These included Luciano et al. (2014; ACT, waitlist and pharmacological treatment), Trompetter et al. (2015; ACT, waitlist and expressive writing), Turner et al. (2016; MBSR, CCBT and TAU), Lin et al. (2017; guided ACT, unguided ACT and waitlist), Pérez‐Aranda et al. (2019; MBSR, TAU and psychoeducation), Hansen et al. (2023; MBSR with ACT, non‐specific intervention and waitlist) and Sanabria‐Mazo, Colomer‐Carbonell, Borràs, et al. (2023; ACT, BATD and TAU). To maintain the independence of observations, inactive and active control arms were pooled into a single control group (e.g., Trompetter et al. 2015; Pérez‐Aranda et al. 2019; and Hansen et al. 2023), intervention arms were pooled when they represented variants of the same approach (Lin et al. 2017), and only comparisons with contextual relevance were retained (Luciano et al. 2014: ACT vs. waitlist; Sanabria‐Mazo, Colomer‐Carbonell, Borràs, et al. 2023: ACT vs. TAU; Turner et al. 2016: MBSR vs. TAU). These decisions were consistently applied across analyses in accordance with Cochrane recommendations (Higgins et al. 2024).

#### 3.5.2 Main Effects

**FIGURE 3:**
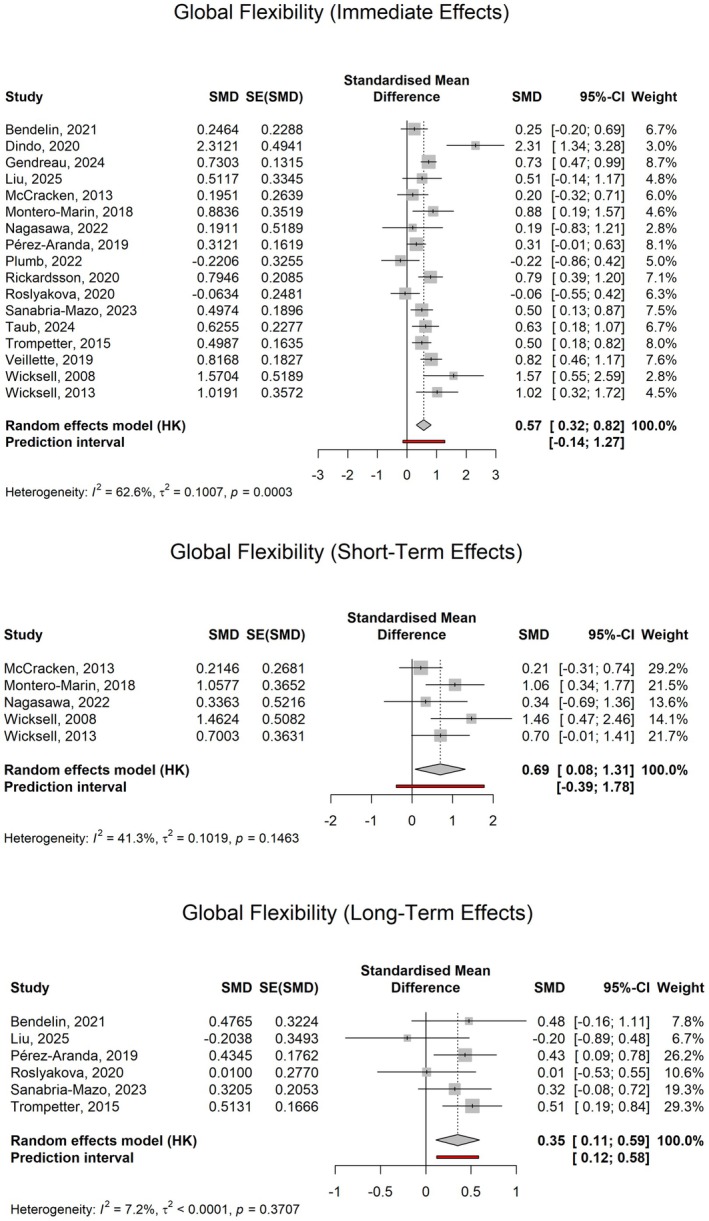
Forest plots of meta‐analytic effects on global psychological flexibility across immediate, short‐term and long‐term follow‐up.

**FIGURE 4:**
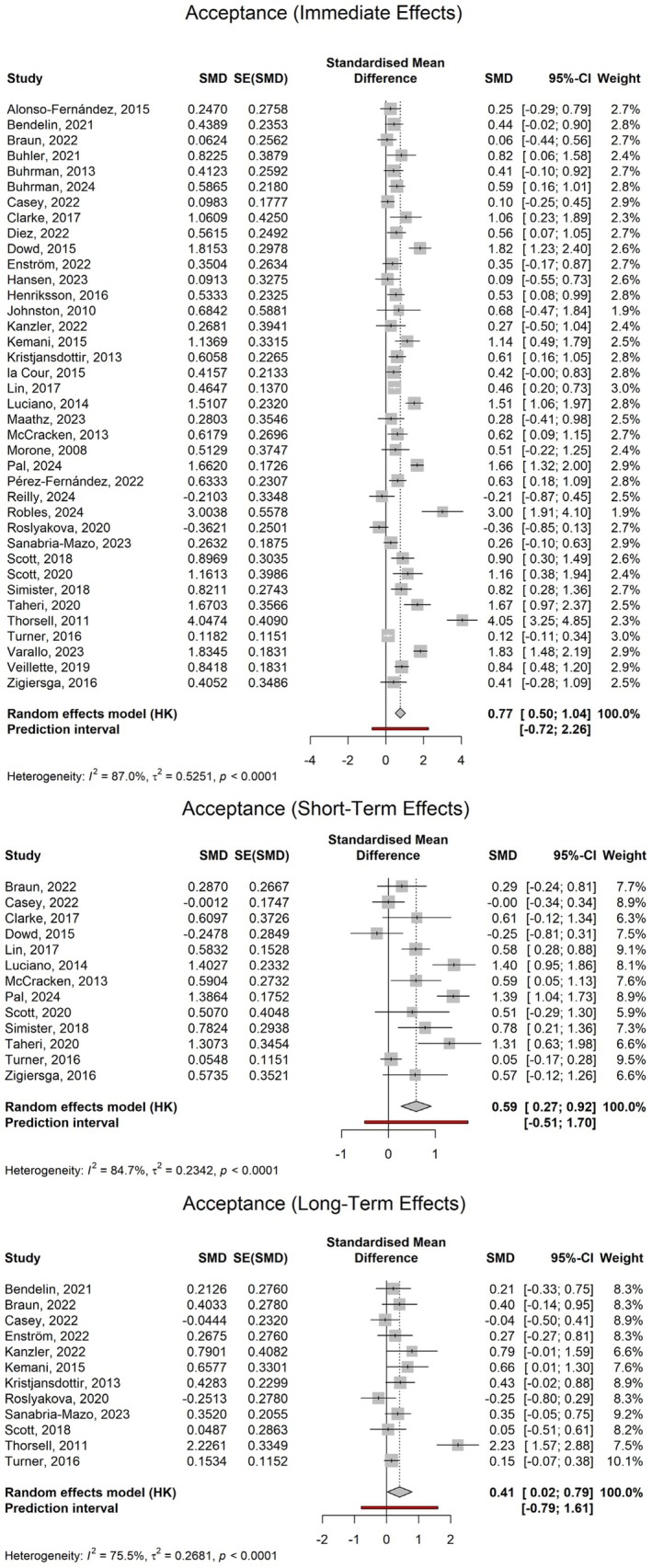
Forest plots of meta‐analytic effects on acceptance across immediate, short‐term and long‐term follow‐up.

**FIGURE 5:**
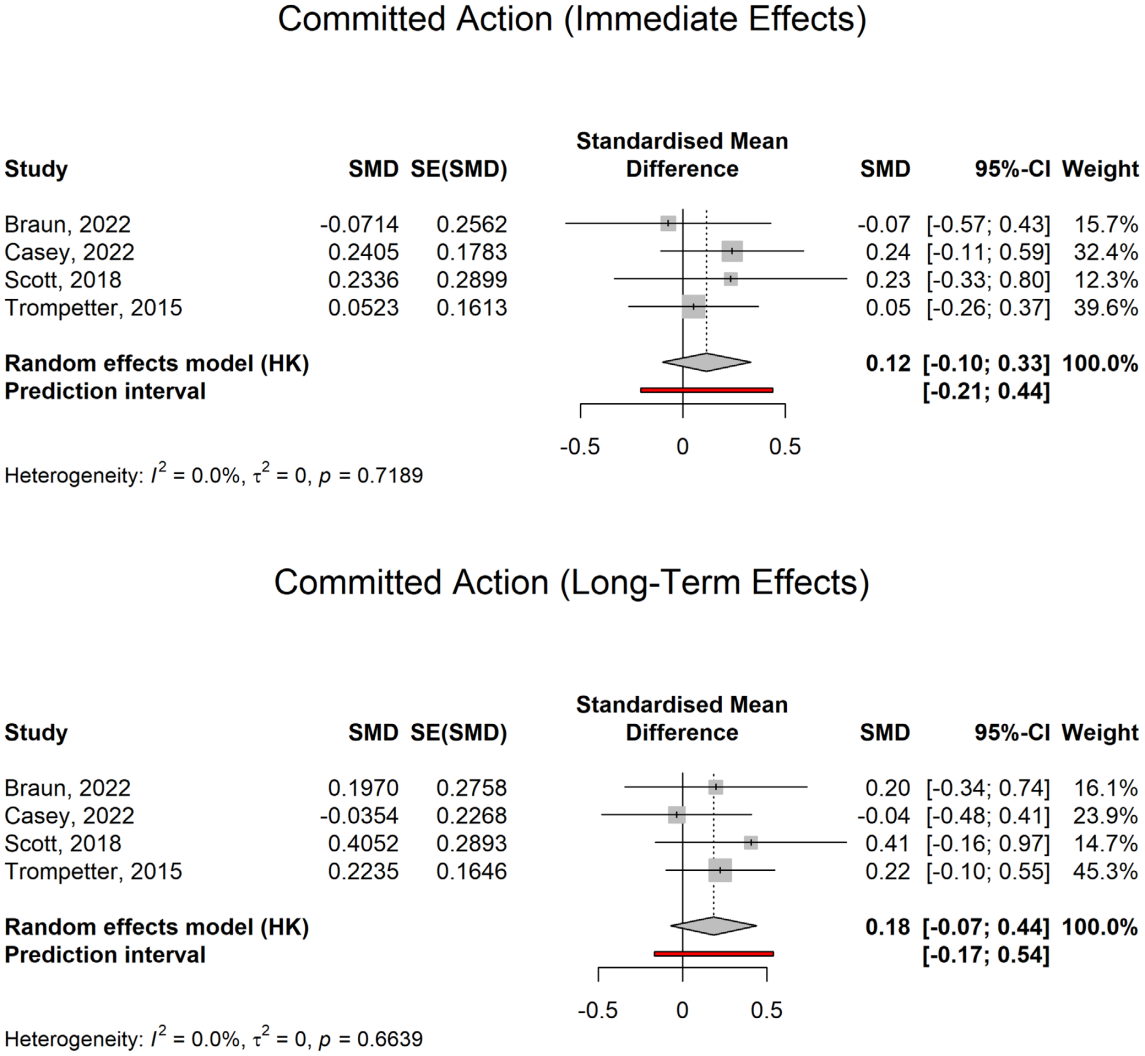
Forest plots of meta‐analytic effects on committed action across immediate and long‐term follow‐up.

**FIGURE 6:**
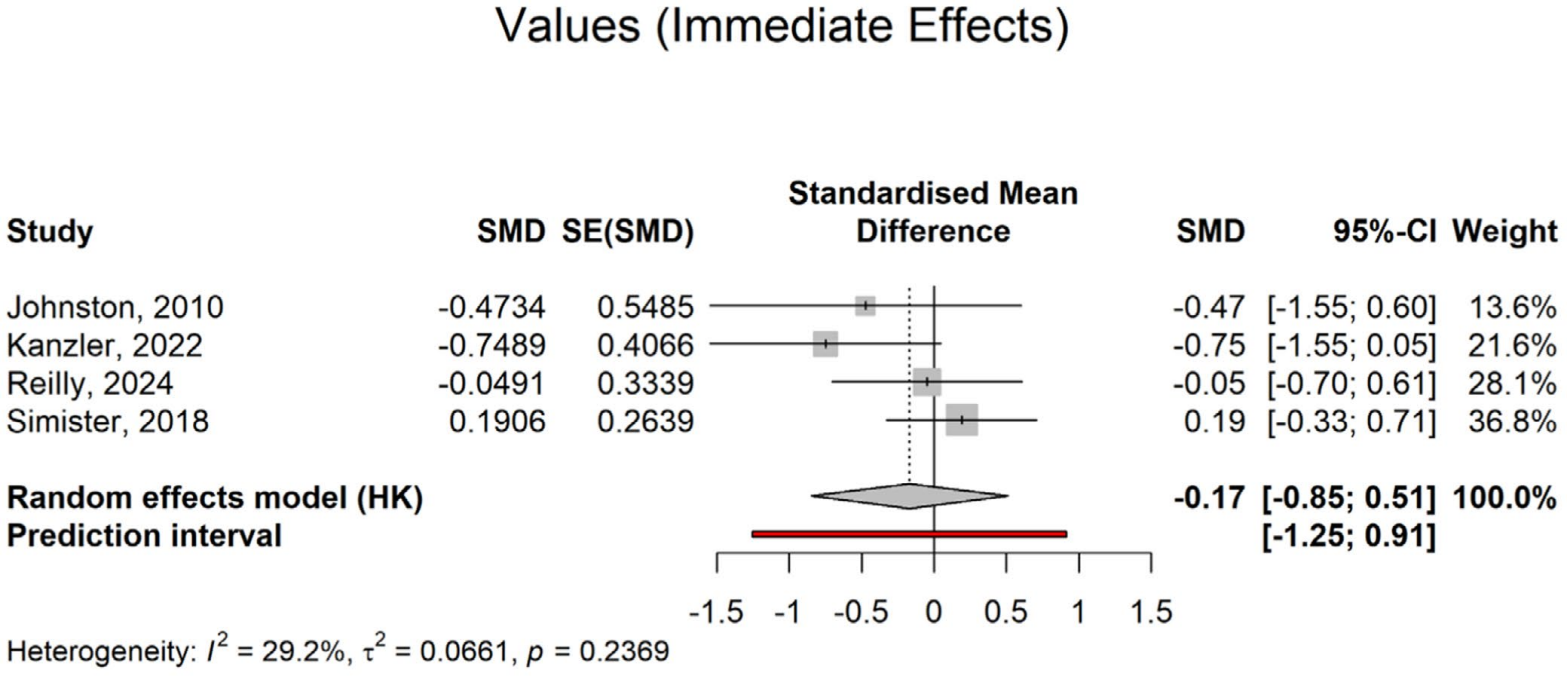
Forest plot of meta‐analytic effects on values‐based action at immediate posttreatment.

Meta‐analyses were conducted for psychological flexibility and three of its core dimensions (i.e., acceptance, committed action and values). For psychological flexibility, data were available from 17 studies on immediate effects, 5 on short‐term effects and 6 on long‐term effects. For acceptance, 38 studies contributed to immediate effects, 13 to short‐term effects and 12 to long‐term effects. For committed action, 4 studies contributed to immediate effects, 2 to short‐term effects and 4 to long‐term effects. For values, 4 studies provided data on immediate effects, 1 on short‐term effects and 1 on long‐term effects. Across assessment periods, psychological flexibility was measured using the PIPS (immediate: *k* = 12; short‐term: *k* = 3; long‐term: *k* = 5) and AAQ‐II (immediate: *k* = 5; short‐term: *k* = 2; long‐term: *k* = 1). Acceptance was measured using the CPAQ (immediate: *k* = 38; short‐term: *k* = 13; long‐term: *k* = 12). Committed action was measured using the CAQ (immediate: *k* = 3; short‐term: *k* = 2; long‐term: *k* = 3) and ELS (immediate: *k* = 1; long‐term: *k* = 1), whereas values were measured using the CPVI (immediate: *k* = 3; long term, *k* = 1) and VQ (immediate: *k* = 1; short‐term: *k* = 1). Evidence was insufficient to support meta‐analyses for cognitive defusion, present‐moment awareness, and self‐as‐context. Forest plots present pooled effect sizes (Hedges' *g*) with 95% confidence intervals for each variable and time point. As shown in Figures 3, 4, 5, 6, positive values indicate greater improvements in the intervention group relative to the control group, whereas negative values indicate greater improvements in the control group.

Certainty of evidence across outcomes is summarized in Table 3. For psychological flexibility, certainty was low at post‐treatment due to risk of bias and inconsistency; very low at short‐term due to risk of bias, imprecision, and publication bias; and low at long‐term due to risk of bias and imprecision. Acceptance was low at post‐treatment and short‐term due to risk of bias and inconsistency, and very low at long‐term due to risk of bias, inconsistency, and imprecision. Committed action and values were consistently low at all time points due to risk of bias and imprecision.

**TABLE 3:** Summary of GRADE evidence for acceptance‐ and mindfulness‐based interventions in adults with chronic pain.

Outcomes	Probable outcome with intervention	No of participants (studies)	Quality of the evidence (GRADE)
Psychological flexibility at post‐treatment PIPS, AAQ‐II Higher scores indicate lower psychological flexibility	The mean psychological flexibility in the intervention groups was **0.57** SDs higher (95% CI: 0.317 to 0.819)	1432 participants (17 studies)	⊕ ⊕ ⊝⊝ Low^a^, ^b^
Psychological flexibility at short term PIPS, AAQ‐II Higher scores indicate lower psychological flexibility	The mean psychological flexibility in the intervention groups was **0.69** SDs higher (95% CI: 0.079 to 1.308)	159 participants (5 studies)	⊕⊝⊝⊝ Very low^a^, ^c^, ^d^
Psychological flexibility at long term PIPS, AAQ‐II Higher scores indicate lower psychological flexibility	The mean psychological flexibility in the intervention groups was **0.35** SDs higher (95% CI: 0.111 to 0.592)	539 participants (6 studies)	⊕ ⊕ ⊝⊝ Low^a^, ^c^
Acceptance at post‐treatment CPAQ Higher scores indicate higher acceptance	The mean acceptance in the intervention groups was **0.77** SDs higher (95% CI: 0.502 to 1.036)	2968 participants (38 studies)	⊕ ⊕ ⊝⊝ Low^a^, ^b^
Acceptance at short term CPAQ Higher scores indicate higher acceptance	The mean acceptance in the intervention groups was **0.59** SDs higher (95% CI: 0.271 to 0.918)	1258 participants (13 studies)	⊕ ⊕ ⊝⊝ Low^a^, ^b^
Acceptance at long term CPAQ Higher scores indicate higher acceptance	The mean acceptance in the intervention groups was **0.41** SDs higher (95% CI: 0.021 to 0.793)	976 participants (12 studies)	⊕⊝⊝⊝ Very low^a^ ^,^ ^b^ ^,^ ^c^
Committed action at post‐treatment ELS, CAQ Higher scores indicate higher committed action	The mean committed action in the intervention groups was 0.116 SDs higher (95% CI: −0.1 to 0.332)	409 participants (4 studies)	⊕ ⊕ ⊝⊝ Low^a^, ^c^
Committed action at long‐term ELS, CAQ Higher scores indicate higher committed action	The mean committed action in the intervention groups was 0.184 SDs higher (95% CI: –0.072 to 0.44)	346 participants (4 studies)	⊕ ⊕ ⊝⊝ Low^a^, ^c^
Values at post‐treatment CPVI, VLQ Higher scores indicate greater alignment with personal values	The mean values score in the intervention groups was 0.17 SDs higher (95% CI: –0.849 to 0.51)	134 participants (4 studies)	⊕ ⊕ ⊝⊝ Low^a^, ^c^

*Note:* Significant SDs (*p* < 0.05) are shown in bold. Population: adults with chronic pain. Settings: community, primary, secondary or tertiary care. Intervention: acceptance‐ and mindfulness‐based interventions. Comparison: active or inactive control. GRADE Working Group levels for the certainty of evidence. High: High confidence that the true effect is close to the estimate. Moderate: Moderate confidence; the true effect may be somewhat different. Low: Low confidence; the true effect may be substantially different. Very low: Very low confidence; the true effect is likely very different.

Abbreviations: AAQ‐II, Acceptance and Action Questionnaire–II; CAQ, Committed Action Questionnaire; CI, confidence interval; CPAQ, Chronic Pain Acceptance Questionnaire; CPVI, Chronic Pain Values Questionnaire; ELS, Engaged Living Scale; PIPS, Psychological Inflexibility in Pain Scale; SD, standard deviation; VLQ, Valued Living Questionnaire.

^a^ Downgraded once for risk of bias.

^b^ Downgraded once for inconsistency.

^c^ Downgraded once for imprecision.

^d^ Downgraded once for publication bias.

Acceptance‐ and MBIs significantly improved psychological flexibility and acceptance relative to control conditions over time. Psychological flexibility showed significant immediate (*g* = 0.57, *p* < 0.001), short‐term (*g* = 0.69, *p* = 0.035) and long‐term (*g* = 0.35, *p* = 0.013) effects. Similarly, acceptance exhibited significant immediate (*g* = 0.77, *p* < 0.001), short‐term (*g* = 0.59, *p* = 0.002) and long‐term (*g* = 0.41, *p* = 0.041) effects. However, no significant immediate (*g* = 0.12, *p* = 0.19) or long‐term effects (*g* = 0.18, *p* = 0.11) were observed for committed action, and no significant immediate effects were found for values (*g* = −0.17, *p* = 0.49).

Heterogeneity across outcomes ranged from low to high. Psychological flexibility showed significant moderate heterogeneity for immediate effects (*I* ^2^ = 62.6%, *p* < 0.001), but was low to moderate and non‐significant at both short‐term (*I* ^2^ = 41.3%, *p* = 0.15) and long‐term (*I* ^2^ = 7.2%, *p* = 0.37) follow‐ups. Acceptance showed high significant heterogeneity across time points (*I* ^2^ = 75.5%–87%, all *p* < 0.001). Committed action and values showed negligible to low non‐significant heterogeneity (*I* ^2^ = 0%–29.2%, *p* > 0.05). Between‐study variance was moderate to high across outcomes (*τ* ^*2*^ = 0.08–0.53), and the corresponding prediction intervals (*g* = −0.10 to 2.30) suggest that negative effects cannot be ruled out in future studies.

Publication bias was examined only for the immediate effects of global psychological flexibility and acceptance, as Egger's test is recommended only when the number of studies is at least 10 (Sterne et al. 2011). Egger's tests provided no evidence of funnel plot asymmetry. Funnel plots and detailed Egger's test statistics are presented in Data S3.

### 3.6 Moderator Analysis

Moderation analyses were conducted only when significant heterogeneity (*p* < 0.10) was detected and when at least 10 studies were available for the overall moderator analysis. Accordingly, analyses were performed only for the immediate effects of psychological flexibility and acceptance. The specific effect sizes for the categorical moderators (i.e., control condition type, intervention type, delivery mode, treatment format, therapist involvement, pain diagnosis, intervention provider, intervention structure and instrument type) are reported in Tables 4 and 5, and for the continuous moderators (i.e., intervention duration, session duration, session frequency, dropout rate, proportion of females and age) in Table 6. Instrument type was examined only for global psychological flexibility, as all meta‐analysed acceptance studies used the CPAQ.

**TABLE 4:** Categorical moderators of psychological flexibility (immediate effects).

Moderators	Sample		Effect size			Heterogeneity		
	*k*	*n*	*g*	95% CI	*p*	*I* ^2^ (%)	*Q*	*p*
Overall	17	1432	0.57	0.317 to 0.819	**< 0.001**	62.6	42.82	**< 0.001**
Control condition type							4.06	**0.044**
Inactive	8	571	0.84	0.363 to 1.326	**0.004**	64.4	19.68	
Active	9	861	0.38	0.121 to 0.634	**0.009**	51.3	16.44	
Intervention type							0	0.951
ACT	14	1142	0.58	0.259 to 0.891	**0.002**	67.1	4	
MBI	3	290	0.56	−0.134 to 1.257	0.074	28.5	0	
Delivery mode							3.31	0.191
Face‐to‐face	10	552	0.61	0.164 to 1.053	**0.013**	69.5	29.5	
Internet‐based	6	842	0.61	0.379 to 0.831	**< 0.001**	19.3	6.19	
Blended/other^a^	1	38	−0.22	−1.097 to 0.656	0.622	—	0	
Treatment format							0.01	0.909
Individual	8	799	0.57	0.226 to 0.921	**0.006**	57.5	16.48	
Group	9	633	0.6	0.142 to 1.061	**0.017**	67.7	24.8	
Therapist involvement							3.26	0.196
Therapist‐delivered	13	783	0.51	0.157 to 0.86	**0.008**	65.5	34.77	
Self‐guided	3	477	0.78	0.664 to 0.893	**0.001**	0	0.17	
Mixed^a^	1	172	0.5	−0.229 to 1.226	0.179	—	0	
Pain diagnosis							3.56	0.169
Unspecified chronic pain	8	653	0.63	0.091 to 1.176	**0.028**	76.6	29.85	
Localized musculoskeletal pain	4	206	0.28	−0.287 to 0.839	0.217	25	4	
Fibromyalgia/other widespread pain	5	573	0.67	0.341 to 1.003	**0.005**	34.7	6.13	
Intervention provider							9.25	**0.01**
Psychologist	14	1132	0.61	0.327 to 0.887	**< 0.001**	62.7	34.85	
Non‐psychologist	2	53	−0.07	−2.577 to 2.427	0.769	0	0.45	
None^a^	1	247	0.73	0.08 to 1.381	**0.028**	—	0	
Intervention structure							4.53	**0.033**
Stand‐alone	14	1252	0.64	0.364 to 0.907	**< 0.001**	61.7	33.95	
Combined	3	180	0.2	−0.481 to 0.888	0.329	1.4	2.03	
Instrument type							0.08	0.772
AAQ‐II	5	199	0.68	−0.476 to 1.841	0.177	80.8	20.87	
PIPS	12	1233	0.56	0.349 to 0.767	**< 0.001**	49.4	21.74	

*Note:* Significant differences (*p* < 0.05) are shown in bold.

Abbreviations: AAQ‐II, Acceptance and Action Questionnaire–II; ACT, acceptance and commitment therapy; MBI, mindfulness‐based intervention; PIPS, Psychological Inflexibility in Pain Scale.

^a^ Additional sensitivity analyses were conducted for the moderators delivery mode, therapist involvement and intervention provider. The subgroup marked with an asterisk was excluded from these analyses.

**TABLE 5:** Categorical moderators of acceptance (immediate effects).

Moderators	Sample		Effect size			Heterogeneity		
	*k*	*n*	*g*	95% CI	*p*	*I* ^2^ (%)	*Q*	*p*
Overall	38	2968	0.77	0.502 to 1.036	**< 0.001**	87	285.05	**< 0.001**
Control condition type							0.3	0.585
Inactive	21	1710	0.69	0.413 to 0.957	**< 0.001**	75.3	80.9	
Active	17	1258	0.84	0.315 to 1.36	**0.004**	91.6	190.2	
Intervention type							0.09	0.764
ACT	27	1932	0.74	0.42 to 1.068	**< 0.001**	86.4	5	
MBI	11	1036	0.83	0.273 to 1.392	**0.008**	89.4	1	
Delivery mode							2.25	0.324
Face‐to‐face	19	1602	0.78	0.404 to 1.154	**< 0.001**	89.7	174.08	
Internet‐based	16	1250	0.58	0.334 to 0.821	**< 0.001**	61	38.48	
Blended/other^a^	3	116	1.94	−2.855 to 6.726	0.224	94.9	39.26	
Treatment format							0.01	0.996
Individual	18	1247	0.76	0.316 to 1.202	**0.002**	84.1	107.15	
Group	19	1692	0.78	0.402 to 1.156	**< 0.001**	89.9	177.71	
Combined^a^	1	29	0.82	−0.834 to 2.478	**0.034**	—	0	
Therapist involvement							0.46	0.794
Therapist‐delivered	31	2389	0.78	0.469 to 1.1	**< 0.001**	88.5	259.76	
Self‐guided	5	321	0.74	−0.17 to 1.655	0.087	81.9	22.1	
Mixed^a^	2	258	0.63	−1.635 to 2.886	0.176	0	0.76	
Pain diagnosis							0.02	0.99
Unspecified chronic pain	21	1461	0.76	0.351 to 1.167	**< 0.001**	85.8	140.91	
Localized musculoskeletal pain	9	878	0.8	0.135 to 1.468	**0.024**	90	80.07	
Fibromyalgia/other widespread pain	8	629	0.79	0.273 to 1.307	**0.009**	86	50.1	
Intervention provider							0.43	0.807
Psychologist	28	2159	0.73	0.425 to 1.043	**< 0.001**	86.8	205.25	
Non‐psychologist	8	667	0.8	0.012 to 1.591	**0.047**	88.7	61.97	
None^a^	2	142	1.16	−6.985 to 9.298	0.322	91.3	11.51	
Intervention structure							22.2	**< 0.001**
Stand‐alone	32	2265	0.9	0.606 to 1.198	**< 0.001**	85.6	215.03	
Combined	6	703	0.11	−1.104 to 0.328	0.242	13.4	5.78	

*Note:* Significant differences (*p* < 0.05) are shown in bold.

Abbreviations: ACT, acceptance and commitment therapy; MBI, mindfulness‐based intervention.

^a^ Additional sensitivity analyses were conducted for the moderators delivery mode, treatment format, therapist involvement and intervention provider. The subgroup marked with an asterisk was excluded from these analyses.

**TABLE 6:** Continuous moderators of psychological flexibility and acceptance (immediate effects).

Moderators	Sample		Effect size			Heterogeneity		
	*k*	*n*	*β*	*95% CI*	*p*	*I* ^2^ (%)	*QE*	*p*
Psychological flexibility								
Intervention duration (weeks)	17	1432	−0.027	−0.108 to 0.053	0.480	69.7	42.77	**< 0.001**
Session duration (min)	15	1085	0.003	−0.001 to 0.007	0.133	66.6	34.13	**0.001**
Session frequency (number of sessions)	17	1432	−0.015	−0.102 to 0.072	0.714	69.5	42.53	**< 0.001**
Dropout rates (%)	17	1432	0.004	−0.027 to 0.035	0.768	68.2	42.82	**< 0.001**
Proportion of females (%)	17	1432	−0.012	−0.025 to 0.002	0.077	64.9	39.3	**< 0.001**
Age (years)	17	1432	−0.022	−0.048 to 0.003	0.083	62.5%	37.28	**0.001**
Acceptance								
Intervention duration (weeks)	38	2968	−0.091	−0.198 to 0.016	0.094	89	234.28	**< 0.001**
Session duration (min)	30	2576	−0.001	0.007 to 0.004	0.691	92.2	257.97	**< 0.001**
Session frequency (number of sessions)	38	2968	−0.038	−0.146 to 0.07	0.485	89.9	259.03	**< 0.001**
Dropout rates (%)	37	2627	0.008	−0.03 to 0.014	0.486	68.2	42.82	**< 0.001**
Proportion of females (%)	37	2933	−0.001	−0.013 to 0.012	0.922	90.4	284.53	**< 0.001**
Age (years)	38	2968	0.001	−0.022 to 0.024	0.916	90.1	284.94	**< 0.001**

*Note:* Significant differences (*p* < 0.05) are shown in bold.

Abbreviations: ACT, acceptance and commitment therapy; MBI, mindfulness‐based intervention.

#### 3.6.1 Categorical Moderators

##### 3.6.1.1 Control Condition Type

For psychological flexibility, the effects of the interventions differed significantly by control condition (*Q*_1_ = 4.06, *p* = 0.044). RCTs comparing interventions with inactive controls showed larger effects (*g* = 0.84) than those with active controls (*g* = 0.38). In contrast, no significant subgroup differences were found for acceptance. The effects did not differ between active and inactive control conditions (*Q*_1_ = 0.30, *p* = 0.59; *g* = 0.68 and 0.84).

##### 3.6.1.2 Intervention Type

No moderating effects were identified in psychological flexibility between ACT and MBIs (*Q*_1_ = 0.01, *p* = 0.95). The effects observed in ACT (*g* = 0.58) and MBIs (*g* = 0.56) were comparable. A similar pattern was observed for acceptance, with no evidence of moderation by intervention type. The effects did not differ between ACT and MBIs (*Q*_1_ = 0.09, *p* = 0.76; *g* = 0.74 and 0.83).

##### 3.6.1.3 Delivery Mode

The impact of delivery mode on psychological flexibility was not significant (*Q*_2_ = 3.31, *p* = 0.19). Effects were comparable across face‐to‐face (*g* = 0.61), internet‐based (*g* = 0.61) and phone‐based (*g* = −0.22) formats. A complementary sensitivity analysis of delivery mode, excluding the phone‐based format study (*k* = 1), showed consistent non‐significant results (*Q*_1_ = 0.05, *p* = 0.83; *g* = 0.67 and 0.62). Regarding acceptance, the delivery mode did not moderate intervention effects. Effect sizes were similar for face‐to‐face, internet‐based and blended/other formats (*Q*_2_ = 2.25, *p* = 0.32; *g* = 0.78, 0.58 and 1.93). A sensitivity analysis excluding blended/other formats yielded the same non‐significant results (*Q*_1_ = 1.02, *p* = 0.31; *g* = 0.78 and 0.57).

##### 3.6.1.4 Treatment Format

No moderating effects of treatment format were found on psychological flexibility (*Q*_1_ = 0.01, *p* = 0.91). Interventions delivered individually (*g* = 0.57) and those implemented in groups (*g* = 0.60) yielded comparable effects. For acceptance, no significant moderating effects were found across the three treatment formats. Effect sizes were similar for individual, group and combined formats (*Q*_2_ = 0.01, *p* = 0.99; *g* = 0.76, 0.78 and 0.82). A sensitivity analysis excluding the combined‐format study (*k* = 1) yielded the same non‐significant results (*Q*_1_ = 0.01, *p* = 0.96; *g* = 0.76 and 0.77).

##### 3.6.1.5 Therapist Involvement

For psychological flexibility, no moderating effect of therapist involvement was identified (*Q*_2_ = 3.26, *p* = 0.20). Effect sizes were comparable across therapist‐delivered (*g* = 0.51), self‐guided (*g* = 0.78) and blended interventions (*g* = 0.50). A sensitivity analysis excluding the blended‐format study (*k* = 1) yielded a consistent non‐significant difference (*Q*_1_ = 2.73, *p* = 0.10; *g* = 0.51 and 0.78). Likewise, acceptance did not significantly differ by therapist involvement. Effect sizes were similar for therapist‐delivered, self‐guided and blended interventions (*Q*_2_ = 0.46, *p* = 0.79; *g* = 0.78, 0.74 and 0.63). A sensitivity analysis excluding the blended subgroup (*k* = 2) yielded the same non‐significant difference (*Q*_1_ = 0.01, *p* = 0.91; *g* = 0.79 and 0.74).

##### 3.6.1.6 Pain Diagnosis

Moderator analyses revealed no significant differences across pain diagnoses (*Q*_*2*_ = 3.56, *p* = 0.17). Effect sizes were comparable for unspecified chronic pain (*g* = 0.63), localized musculoskeletal pain (*g* = 0.28) and fibromyalgia or other widespread pain (*g* = 0.67). Similarly, acceptance did not vary by the type of pain diagnosis. Effects were similar for unspecified chronic pain, localized musculoskeletal pain and fibromyalgia or other widespread pain (*Q*_2_ = 0.02, *p* = 0.99; *g* = 0.76, 0.80 and 0.79).

##### 3.6.1.7 Intervention Provider

Provider type significantly moderated outcomes for psychological flexibility (*Q*_2_ = 9.25, *p* = 0.010). Interventions delivered by psychologists (*g* = 0.61) or interventions without therapist involvement (*g* = 0.73) showed larger effects than those delivered by non‐psychologists (*g* = −0.07). A sensitivity analysis excluding the self‐guided intervention (*k* = 1) yielded the same significant pattern (*Q*_1_ = 8.36, *p* = 0.004; *g* = 0.61 and −0.01). In contrast, provider type did not moderate the intervention's effects on acceptance. Effect sizes were similar for psychologist‐, non‐psychologist– and self‐guided interventions (*Q*_*2*_ = 0.43, *p* = 0.81; *g* = 0.73, 0.80 and 1.16). A sensitivity analysis excluding the self‐guided subgroup (*k* = 2) yielded consistent results (*Q*_1_ = 0.04, *p* = 0.83; *g* = 0.73 and 0.81).

##### 3.6.1.8 Intervention Structure

Intervention structure significantly moderated the effects on psychological flexibility (*Q*_1_ = 4.53, *p* = 0.033). Effect sizes were larger for stand‐alone interventions (*g* = 0.64) than for combined ones (*g* = 0.20). For acceptance, a significant moderation effect was also observed. Effect sizes were larger for stand‐alone than for combined interventions (*Q*_1_ = 22.20, *p* < 0.001; *g* = 0.90 and 0.11).

##### 3.6.1.9 Instrument Type

For psychological flexibility, instrument type did not significantly moderate intervention effects (*Q*_1_ = 0.08, *p* = 0.772). Effects were comparable between studies using the AAQ‐II (*g* = 0.68) and the PIPS (*g* = 0.56), although heterogeneity was higher in studies using the AAQ‐II (*I* ^2^ = 80.8%) than in those using the PIPS (*I* ^2^ = 49.4%). This analysis was not conducted for acceptance because all meta‐analysed studies used the CPAQ.

#### 3.6.2 Continuous Moderators

None of the continuous variables examined (intervention duration, session duration, session frequency, dropout rate, proportion of females or age) significantly moderated the effects on either psychological flexibility or acceptance. Detailed results are provided in Table 6.

## 4 Discussion

This review synthesized the effects of acceptance‐ and MBIs on psychological flexibility in people with chronic pain. Sixty RCTs were included in the systematic review, of which 51 contributed to the meta‐analysis. These interventions were associated with significant improvements in psychological flexibility and acceptance compared with control conditions, with small‐to‐medium effects that were maintained over time. However, these findings require caution because most trials were at high risk of bias, the certainty of evidence was low to very low and heterogeneity was substantial. Evidence for values and committed action was limited and non‐significant, and evidence was insufficient to meta‐analyse cognitive defusion, present‐moment awareness and self‐as‐context. Therefore, evidence remains uneven across flexibility dimensions, limiting conclusions beyond global psychological flexibility and acceptance.

Previous meta‐analyses in chronic pain have shown that acceptance‐ and MBIs reduce pain intensity, depression, anxiety, disability, and pain‐interference and improve quality of life (Hilton et al. 2017; Lai et al. 2023; Martinez‐Calderon et al. 2024; Veehof et al. 2016). However, these reviews have focused on clinical outcomes, and none have examined psychological flexibility and its dimensions. Understanding this process is clinically relevant because it indicates that these treatments produce theoretically consistent and specific effects. Taken together with evidence that psychological flexibility is associated with pain‐related outcomes and treatment response (Ding and Zheng 2022; Fang and Ding 2022; McCracken 2024; McCracken and Morley 2014; McCracken and Vowles 2014), this convergence is clinically meaningful.

Moderator analyses were limited to immediate effects on global psychological flexibility and acceptance. For global psychological flexibility and acceptance, stand‐alone interventions showed greater and more consistent effects than combined approaches. This finding is consistent with some studies (Dragioti et al. 2018; Kamper et al. 2014), but contrasts with others (Häuser et al. 2009; Thieme et al. 2017). These findings may suggest that focusing on a single targeted component is associated with larger effects, although moderator results should be interpreted with caution, as they may reflect methodological or study‐level differences rather than true clinical differences.

No significant differences in intervention effects were found by pain type, in line with the transdiagnostic nature of psychological flexibility and with evidence that psychological factors tend to be similar across chronic pain conditions (Burke et al. 2015; Fillingim et al. 2020). This suggests applicability across chronic pain populations. In addition, both ACT and MBI showed comparable effects, consistent with previous research suggesting that psychological flexibility may serve as an underlying process in both approaches (Gu et al. 2015; Scott et al. 2016). This finding, however, contrasts with meta‐analyses reporting greater effectiveness of these interventions for localized pain than for non‐specific pain (Lai et al. 2023; Ma et al. 2023).

No significant differences were observed in delivery mode, treatment format or level of support for global psychological flexibility and acceptance, suggesting effects across implementation formats. This differs from previous evidence in chronic pain (Alldredge et al. 2023; Lai et al. 2023; Mehta et al. 2019), where face‐to‐face delivery, group‐based formats, and therapist‐guided programmes have been associated with larger effects on clinical outcomes such as pain intensity, disability or psychological distress rather than process‐related outcomes. It is possible that clinical outcomes are strongly shaped by a combination of common therapeutic factors, such as therapeutic alliance, interpersonal support or treatment context, and intervention‐specific processes, whereas changes in psychological flexibility may be closely tied to how acceptance‐ and mindfulness‐based strategies are implemented across formats. This suggests that effects on psychological flexibility and acceptance may be comparable across formats.

Regarding the provider type, significant differences were found for global psychological flexibility. Psychologist‐delivered interventions showed larger effects than those delivered by non‐psychologists. This result is supported by evidence indicating that psychological interventions tend to yield better clinical outcomes when delivered by clinicians with specialized psychological training (Bostick 2017; Cuijpers et al. 2021; Del Re et al. 2021; Frank et al. 2020). Nevertheless, psychologist‐delivered interventions improved global psychological flexibility but not acceptance, suggesting that their added value may be domain‐specific rather than universal. A plausible explanation is that psychologists typically receive more training in behavioural and contextual therapeutic skills, which may be relevant for enhancing global psychological flexibility. The type of control condition also emerged as a significant moderator for global psychological flexibility but not for acceptance, with inactive control groups showing larger effects.

Continuous moderators did not significantly predict effect sizes for psychological flexibility or its dimensions. Neither intervention duration, session length, dropout rates, proportion of females, nor participants' age was associated with treatment effects, indicating that changes in psychological flexibility are consistent across a wide range of intervention characteristics and sample compositions. This finding differs from previous meta‐analyses reporting that longer treatment duration is associated with improved outcomes, although those studies did not specifically examine psychological flexibility (Ma et al. 2023; Veehof et al. 2011). These results suggest that improvements in flexibility‐related processes may depend less on structural or demographic factors and more on the therapeutic strategies employed.

### 4.1 Future Recommendations and Implications

Future studies should test psychological flexibility as a mediator of treatment effects on clinical outcomes such as disability, pain intensity, psychological distress and quality of life. This is important because psychological flexibility was often assessed as a secondary or process‐related outcome rather than as a primary target of evaluation, which may reflect the continued emphasis of chronic pain trials on clinical outcomes over mechanisms of change. Identifying which components of acceptance‐ and mindfulness‐based therapies most effectively enhance psychological flexibility, and for whom, may be informative. This may inform more process‐based and individualized interventions. From a practical perspective, ACT and MBI showed comparable effectiveness, with no differences between in‐person and online formats. This suggests that brief online programs incorporating acceptance‐based practices may represent a scalable option. Interventions delivered by psychologists showed stronger effects for global psychological flexibility, highlighting the value of specialized psychological training.

### 4.2 Limitations and Strengths

Several limitations should be acknowledged. Heterogeneity was substantial, particularly for acceptance, suggesting that differences in intervention content, control conditions, populations and measurement approaches may have contributed to variability in effect estimates. The risk of bias was high in most included trials. As is common in psychological research, bias in outcome measurement was rated as high because outcomes were self‐reported, evaluators were often not blinded to group assignment and psychological measures are often subjective. According to the RoB algorithm, a single high‐risk domain yields an overall risk rating. Therefore, the global risk‐of‐bias rating should be interpreted with caution, particularly given the methodological constraints of psychological intervention trials, where some blinding criteria are difficult to satisfy (Munder and Barth 2018). Consistent with this, the GRADE assessment rated the certainty of the evidence as low to very low across outcomes, primarily due to the high risk of bias.

In addition, the small number of eligible studies prevented meta‐analyses for the flexibility dimensions of cognitive defusion, present‐moment awareness and self‐as‐context. This limits conclusions about specific dimensions of the hexaflex model, even though broader measures of global psychological flexibility and acceptance may capture clinically relevant flexibility‐related processes. Moderator analyses also require caution, as some specific subgroups were represented by few studies. The pattern of findings suggests reasonable stability over time; however, long‐term results should be interpreted cautiously because not all studies included long‐term follow‐up assessments.

Although widely used, the AAQ‐II has been criticized for its overlap with general distress and its limited coverage of psychological flexibility, which should be considered when interpreting results from this measure. Conceptual and operational heterogeneity across instruments assessing psychological flexibility may have contributed to between‐study variability. In addition, ongoing debate remains regarding whether psychological flexibility and inflexibility represent opposite ends of a bipolar continuum or partially distinct constructs, which may further complicate the interpretation and aggregation of broad flexibility measures across studies (Lucas et al. 2025; Spencer and Tyndall 2025). This issue is particularly relevant to broad measures such as the AAQ‐II and PIPS, which may capture both overlapping and distinct flexibility‐related processes. Finally, samples were predominantly female, potentially underrepresenting males. While sex did not moderate the main results, future research should address this imbalance to improve generalizability.

Despite these limitations, this is the first systematic review and meta‐analysis to examine psychological flexibility and its dimensions as processes that improve following acceptance‐ and MBIs in people with chronic pain. Among the strengths of the study are the large number of studies included in the meta‐analyses and the substantial total sample size. Additionally, the inclusion of multiple follow‐up time points and the comprehensive assessment of categorical and continuous moderators further strengthen the robustness of the findings.

## 5 Conclusion

This review suggests that acceptance‐ and MBIs may improve psychological flexibility in people with chronic pain. This extends previous evidence on pain‐related clinical outcomes by showing potential effects on process‐related variables, although the overall certainty of the evidence was low. The results were consistent, with larger effects observed when interventions were delivered by psychologists or implemented as stand‐alone programs. These findings cautiously support psychological flexibility as a potentially important treatment‐related process in chronic pain, while underscoring the need for more methodologically rigorous trials and mediation analyses.

## Author Contributions

Juan P. Sanabria‐Mazo and Juan V. Luciano conceived and designed the study. Juan P. Sanabria‐Mazo, Carla Rodríguez‐Freire and Estíbaliz Royuela‐Colomer wrote the first draft of the manuscript. Juan P. Sanabria‐Mazo, Carla Rodríguez‐Freire, Estíbaliz Royuela‐Colomer, Paula Ponce‐López, Valentina Barrios and Jaime Navarrete participated in study selection, risk‐of‐bias assessment, methodological quality evaluation or data extraction. Estíbaliz Royuela‐Colomer analyzed the data. Paula Ponce‐López, Pablo Alonso‐Coello, Valentina Barrios, Jaime Navarrete, Adrián Pérez‐Aranda, Lance M. McCracken and Juan V. Luciano contributed to the final drafting and editing of the manuscript. All authors contributed to the interpretation of the results and to the revision of the manuscript for important intellectual content.

## Funding

Juan P. Sanabria‐Mazo has a Juan de la Cierva postdoctoral contract awarded by the Spanish Ministry of Science (JDC2024‐053318‐I). Carla Rodríguez‐Freire has a research contract from the Institute of Health Carlos III (ISCIII; ICI20/00080). Jaime Navarrete has a postdoctoral contract awarded by the Centre for Biomedical Research in Epidemiology and Public Health (CIBERESP; CB22/02/00052). The authors thank CIBERESP for the financial support (CB22/02/00052 & CB06/02/1010). The ISCIII and CIBERESP did not play any role in the analysis and interpretation of data, in the writing of the manuscript or in the decision to submit the article for publication.

## Disclosure

Use of Artificial Intelligence: Generative artificial intelligence (AI) was not used in the preparation of this manuscript.

## Conflicts of Interest

The authors declare no conflicts of interest.

## Supporting information


**Data S1:** References for studies included in the review.

**Data S2:** Characteristics of the intervention and control conditions of the included RCTs.

**Data S3:** Eggers' test and funnel plots.

**Table S1:** Search strategy.

**Table S2:** Characteristics of excluded studies.

## Data Availability

The data that support the findings of this study are available at: https://osf.io/kb4xr.
