# Supplementary material for: Do Acceptance‐ and Mindfulness‐Based Interventions Improve Psychological Flexibility in People With Chronic Pain? A Systematic Review and Meta‐Analysis of Randomized Controlled Trials
Source: Eur J Pain. 2026 Aug 1;30(7):e70342. doi: 10.1002/ejp.70342 (PMC13428482; doi:10.1002/ejp.70342)
Supplement: Supplementary file 1 — Data S1: References for studies included in the review. [file EJP-30-0-s002.docx]

**Supplementary Material 1. References for Studies Included in the Review**

1. Alonso-Fernández, M., López-López, A., Losada, A., González, J. L., & Wetherell, J. L. (2015). Acceptance and commitment therapy and selective optimization with compensation for institutionalized older people with chronic pain. *Pain Medicine, 17*(2), 264–277. <https://doi.org/10.1111/pme.12885>
2. Bendelin, N., Gerdle, B., Blom, M., Södermark, M., & Andersson, G. (2021). Internet-delivered acceptance and commitment therapy added to multimodal pain rehabilitation: A cluster randomized controlled trial. *Journal of Clinical Medicine, 10*(24), 5872. <https://doi.org/10.3390/jcm10245872>
3. Bostrøm, K., Børøsund, E., Eide, H., Varsi, C., Kristjansdottir, Ó. B., Schreurs, K. M., ... & Solberg Nes, L. (2023). Short-term findings from testing EPIO, a digital self-management program for people living with chronic pain: randomized controlled trial. *Journal of Medical Internet Research*, 25, e47284. <https://doi.org/10.2196/47284>
4. Braun, L., Terhorst, Y., Titzler, I., Freund, J., Thielecke, J., Ebert, D. D., & Baumeister, H. (2022). Lessons learned from an attempted pragmatic randomized controlled trial for improvement of chronic pain-associated disability in green professions: Long-term effectiveness of a guided online-based acceptance and commitment therapy (PACT-A). *International Journal of Environmental Research and Public Health, 19*(21), 13858. <https://doi.org/10.3390/ijerph192113858>
5. Buhler, J. N., Holens, P. L., & Sharpe, D. (2021). A randomized controlled trial of an online acceptance and commitment therapy-based intervention for chronic pain for military and police. *Military Behavioral Health, 9*(4), 463–474. <https://doi.org/10.1080/21635781.2021.1982086>
6. Buhrman, M., Hällström, H., Fridén, A., Kettis Moden, E., Grahn, G., Carlfjord, M., Hjo, C., Gasslander, N., Bohm‐Starke, N., & McCracken, L. M. (2024). Guided internet‐based acceptance and commitment therapy for provoked vestibulodynia: A randomized controlled trial. *European Journal of Pain, 28*(7), 1185–1201. <https://doi.org/10.1002/ejp.2253>
7. Buhrman, M., Skoglund, A., Husell, J., Bergström, K., Gordh, T., Hursti, T., Bendelin, N., Furmark, T., & Andersson, G. (2013). Guided internet-delivered acceptance and commitment therapy for chronic pain patients: A randomized controlled trial. *Behaviour Research and Therapy, 51*(6), 307–315. <https://doi.org/10.1016/j.brat.2013.02.010>
8. Buhrman, M., Syk, M., Burvall, O., Hartig, T., Gordh, T., & Andersson, G. (2015). Individualized guided internet-delivered cognitive-behavior therapy for chronic pain patients with comorbid depression and anxiety: A randomized controlled trial. *The Clinical Journal of Pain, 31*(6), 504–516. <https://doi.org/10.1097/AJP.0000000000000176>
9. Casey, M.-B., Smart, K. M., Segurado, R., Hearty, C., Gopal, H., Lowry, D., Flanagan, D., McCracken, L., & Doody, C. (2022). Exercise combined with acceptance and commitment therapy compared with a standalone supervised exercise programme for adults with chronic pain: A randomised controlled trial. *Pain, 163*(6), 1158–1171. <https://doi.org/10.1097/j.pain.0000000000002487>
10. Clarke, S. P., Poulis, N., Moreton, B. J., Walsh, D. A., & Lincoln, N. B. (2017). Evaluation of a group acceptance commitment therapy intervention for people with knee or hip osteoarthritis: A pilot randomized controlled trial. *Disability and Rehabilitation, 39*(7), 663–670. <https://doi.org/10.3109/09638288.2016.1160295>
11. Diez, G. G., Anitua, E., Castellanos, N., Vázquez, C., Galindo-Villardón, P., & Alkhraisat, M. H. (2022). The effect of mindfulness on the inflammatory, psychological and biomechanical domains of adult patients with low back pain: A randomized controlled clinical trial. *PLOS ONE, 17*(11), e0276734. <https://doi.org/10.1371/journal.pone.0276734>
12. Dindo, L., Johnson, A. L., Lang, B., Rodrigues, M., Martin, L., & Jorge, R. (2020). Development and evaluation of an 1-day acceptance and commitment therapy workshop for veterans with comorbid chronic pain, TBI, and psychological distress: Outcomes from a pilot study. *Contemporary Clinical Trials, 90*, 105954. <https://doi.org/10.1016/j.cct.2020.105954>
13. Dowd, H., Hogan, M. J., McGuire, B. E., Davis, M. C., Sarma, K. M., Fish, R. A., & Zautra, A. J. (2015). Comparison of an online mindfulness-based cognitive therapy intervention with online pain management psychoeducation: A randomized controlled study. *The Clinical Journal of Pain, 31*(6), 517–527. <https://doi.org/10.1097/AJP.0000000000000201>
14. Gasslander, N., Andersson, G., Boström, F., Brandelius, L., Pelling, L., Hamrin, L., Gordh, T., & Buhrman, M. (2022). Tailored internet-based cognitive behavioral therapy for individuals with chronic pain and comorbid psychological distress: A randomized controlled trial. *Cognitive Behaviour Therapy, 51*(5), 408–434. <https://doi.org/10.1080/16506073.2022.2065528>
15. Gendreau, R. M., McCracken, L. M., Williams, D. A., Luciano, J. V., Dai, Y., Vega, N., Ghalib, Z., Guthrie, K., Kraus, A. C., Rosenbluth, M. J., Vaughn, B., Zomnir, J. M., Reddy, D., Chadwick, A. L., Clauw, D. J., & Arnold, L. M. (2024). Self-guided digital behavioural therapy versus active control for fibromyalgia (PROSPER-FM): A phase 3, multicentre, randomised controlled trial. *The Lancet, 404*(10450), 364–374. <https://doi.org/10.1016/S0140-6736(24)00909-7>
16. Godfrey, E., Wileman, V., Galea Holmes, M., McCracken, L. M., Norton, S., Moss-Morris, R., Noonan, S., Barcellona, M., & Critchley, D. (2020). Physical therapy informed by acceptance and commitment therapy (PACT) versus usual care physical therapy for adults with chronic low back pain: A randomized controlled trial. *The Journal of Pain, 21*(1–2), 71–81. <https://doi.org/10.1016/j.jpain.2019.05.012>
17. Hansen, K. E., Brandsborg, B., Kesmodel, U. S., Forman, A., Kold, M., Pristed, R., Donchulyesko, O., Hartwell, D., & Vase, L. (2023). Psychological interventions improve quality of life despite persistent pain in endometriosis: Results of a 3-armed randomized controlled trial. *Quality of Life Research, 32*(6), 1727–1744. <https://doi.org/10.1007/s11136-023-03346-9>
18. Henriksson, J., Wasara, E., & Rönnlund, M. (2016). Effects of eight-week-web-based mindfulness training on pain intensity, pain acceptance, and life satisfaction in individuals with chronic pain. *Psychological Reports, 119*(3), 586–607. <https://doi.org/10.1177/0033294116675086>
19. Herbert, M. S., Afari, N., Liu, L., Heppner, P., Rutledge, T., Williams, K., Eraly, S., VanBuskirk, K., Nguyen, C., Bondi, M., Atkinson, J. H., Golshan, S., & Wetherell, J. L. (2017). Telehealth versus in-person acceptance and commitment therapy for chronic pain: A randomized noninferiority trial. *The Journal of Pain, 18*(2), 200–211. <https://doi.org/10.1016/j.jpain.2016.10.014>
20. Hess Engström, A., Bohm-Starke, N., Kullinger, M., Hesselman, S., Högberg, U., Buhrman, M., & Skalkidou, A. (2022). Internet-based treatment for vulvodynia (EMBLA)–a randomized controlled study. *The Journal of Sexual Medicine, 19*(2), 319-330. <https://doi.org/10.1016/j.jsxm.2021.11.019>
21. incus, T., Anwar, S., McCracken, L. M., McGregor, A., Graham, L., Collinson, M., McBeth, J., Watson, P., Morley, S., Henderson, J., Farrin, A. J., & on behalf of the OBI Trial Management Team. (2015). Delivering an optimised behavioural intervention (OBI) to people with low back pain with high psychological risk; Results and lessons learnt from a feasibility randomised controlled trial of contextual cognitive behavioural therapy (CCBT) vs. physiotherapy. *BMC Musculoskeletal Disorders, 16*(1), 147. <https://doi.org/10.1186/s12891-015-0594-2>
22. Johnston, M., Foster, M., Shennan, J., Starkey, N. J., & Johnson, A. (2010). The effectiveness of an acceptance and commitment therapy self-help intervention for chronic pain. *The Clinical Journal of Pain, 26*(5), 393–402. <https://doi.org/10.1097/AJP.0b013e3181cf59ce>
23. Kanzler, K. E., Robinson, P. J., McGeary, D. D., Mintz, J., Kilpela, L. S., Finley, E. P., McGeary, C., Lopez, E. J., Velligan, D., Munante, M., Tsevat, J., Houston, B., Mathias, C. W., Potter, J. S., & Pugh, J. (2022). Addressing chronic pain with focused acceptance and commitment therapy in integrated primary care: Findings from a mixed methods pilot randomized controlled trial. *BMC Primary Care*, 23(1), 77. <https://doi.org/10.1186/s12875-022-01690-2>
24. Kemani, M. K., Olsson, G. L., Lekander, M., Hesser, H., Andersson, E., & Wicksell, R. K. (2015). Efficacy and cost-effectiveness of acceptance and commitment therapy and applied relaxation for longstanding pain: A randomized controlled trial. *The Clinical Journal of Pain, 31*(11), 1004–1016. <https://doi.org/10.1097/AJP.0000000000000203>
25. Kristjánsdóttir, Ó. B., Fors, E. A., Eide, E., Finset, A., Stensrud, T. L., van Dulmen, S., ... & Eide, H. (2013). A smartphone-based intervention with diaries and therapist-feedback to reduce catastrophizing and increase functioning in women with chronic widespread pain: randomized controlled trial. *Journal of Medical Internet Research, 15*(1), e5. <https://doi.org/10.2196/jmir.2249>
26. La Cour, P., & Petersen, M. (2015). Effects of mindfulness meditation on chronic pain: A randomized controlled trial. *Pain Medicine, 16*(4), 641–652. <https://doi.org/10.1111/pme.12605>
27. Lin, J., Paganini, S., Sander, L., Lüking, M., Ebert, D. D., Buhrman, M., Andersson, G., & Baumeister, H. (2017). An internet-based intervention for chronic pain. *Deutsches Ärzteblatt International*. <https://doi.org/10.3238/arztebl.2017.0681>
28. Liu, J. Q. J., Mak, Y. W., Tang, A. L. Y., Kwan, C., Al Zoubi, F., Wong, T. K. T., Tsang, G. S. H., Kwong, H. C. W., Lai, S. W. T., Sze, S. P. S., Hui, K. T. K., Cheung, C. K. C., Samartzis, D., Chow, K. K. S., & Wong, A. Y. L. (2025). Effects of acceptance and commitment therapy plus exercise for older adults with chronic low back pain: A preliminary cluster randomized controlled trial with qualitative interviews. *The Journal of Pain,* 30, 105350. <https://doi.org/10.1016/j.jpain.2025.105350>
29. Luciano, J. V., Guallar, J. A., Aguado, J., López-del-Hoyo, Y., Olivan, B., Magallón, R., Alda, M., Serrano-Blanco, A., Gili, M., & Garcia-Campayo, J. (2014). Effectiveness of group acceptance and commitment therapy for fibromyalgia: A 6-month randomized controlled trial (EFFIGACT study). *Pain, 155*(4), 693–702. <https://doi.org/10.1016/j.pain.2013.12.029>
30. Maathz, P., McCracken, L. M., Eriksson, V., Säde, F., Aneblom, G., Rikner, Å., Skalkidou, A., & Buhrman, M. (2023). A feasibility trial of online acceptance and commitment therapy for women with provoked vestibulodynia. *Scandinavian Journal of Pain, 23*(3), 476–482. <https://doi.org/10.1515/sjpain-2022-0146>
31. McCracken, L. M., Sato, A., & Taylor, G. J. (2013). A trial of a brief group-based form of acceptance and commitment therapy (ACT) for chronic pain in general practice: Pilot outcome and process results. *The Journal of Pain, 14*(11), 1398–1406. <https://doi.org/10.1016/j.jpain.2013.06.011>
32. Montero-Marín, J., Navarro-Gil, M., Puebla-Guedea, M., Luciano, J. V., Van Gordon, W., Shonin, E., & García-Campayo, J. (2018). Efficacy of “attachment-based compassion therapy” in the treatment of fibromyalgia: A randomized controlled trial. *Frontiers in Psychiatry, 8*, 307. <https://doi.org/10.3389/fpsyt.2017.00307>
33. Morone, N. E., Greco, C. M., & Weiner, D. K. (2008). Mindfulness meditation for the treatment of chronic low back pain in older adults: A randomized controlled pilot study. *Pain, 134*(3), 310–319. <https://doi.org/10.1016/j.pain.2007.04.038>
34. Nagasawa, Y., Shibata, A., Fukamachi, H., Ishii, K., & Oka, K. (2022). Physical therapist-delivered acceptance and commitment therapy and exercise for older outpatients with knee osteoarthritis: A pilot randomized controlled trial. *Journal of Physical Therapy Science, 34(*12), 784–790. <https://doi.org/10.1589/jpts.34.784>
35. Nes, L. S., Børøsund, E., Varsi, C., Eide, H., Waxenberg, L. B., Weiss, K. E., ... & Schreurs, K. M. (2024). Living well with chronic pain: a 12-month randomized controlled trial revealing impact from the digital pain self-management program EPIO. *Pain Reports, 9*(4), e1174. <https://doi.org/10.1097/PR9.0000000000001174>
36. Pal, A., Mukhopadhyay, P., Bhattacharyya, D., & Ray, S. (2024). Effects of a mindfulness-based intervention on pain intensity, disability and quality of life of chronic low back pain patients: A randomised study. *Indian Journal of Anaesthesia, 68*(10), 915–920. <https://doi.org/10.4103/ija.ija_361_24>
37. Pérez-Aranda, A., Feliu-Soler, A., Montero-Marín, J., García-Campayo, J., Andrés-Rodríguez, L., Borràs, X., Rozadilla-Sacanell, A., Peñarrubia-Maria, M. T., Angarita-Osorio, N., McCracken, L. M., & Luciano, J. V. (2019). A randomized controlled efficacy trial of mindfulness-based stress reduction compared with an active control group and usual care for fibromyalgia: The EUDAIMON study. *Pain, 160*(11), 2508–2523. <https://doi.org/10.1097/j.pain.0000000000001655>
38. Pérez-Fernández, J. I., Salaberria, K., & Ruiz De Ocenda, Á. (2022). Mindfulness-based pain management (MBPM) for chronic pain: A randomized clinical trial. *Mindfulness, 13*(12), 3153–3165. <https://doi.org/10.1007/s12671-022-02023-1>
39. Plumb Vilardaga, J. C., Kelleher, S. A., Diachina, A., Riley, J., & Somers, T. J. (2022). Linking physical activity to personal values: feasibility and acceptability randomized pilot of a behavioral intervention for older adults with osteoarthritis pain. *Pilot and Feasibility Studies, 8*(1), 164. <https://doi.org/10.21203/rs.3.rs-1182374/v1>
40. Ramos, C., Ríos, F. L., Muñante, G. P., & Ordóñez-Carrasco, J. (2024). Group acceptance and commitment therapy (ACT) for fibromyalgia patients. *Clínica y Salud, 35*(2), 39–48. <https://doi.org/10.5093/clysa2024a1>
41. Reilly, E. D., Kelly, M. M., Grigorian, H. L., Waring, M. E., Quigley, K. S., Hogan, T. P., Heapy, A. A., Drebing, C. E., Volonte, M., Kathawalla, U.-K., Robins, H. E., Bernice, K., & Bickmore, T. (2024). Virtual coach–guided online acceptance and commitment therapy for chronic pain: Pilot feasibility randomized controlled trial. *JMIR Formative Research, 8*, e56437. <https://doi.org/10.2196/56437>
42. Rickardsson, J., Gentili, C., Holmström, L., Zetterqvist, V., Andersson, E., Persson, J., Lekander, M., Ljótsson, B., & Wicksell, R. K. (2020). Internet‐delivered acceptance and commitment therapy as microlearning for chronic pain: A randomized controlled trial with 1‐year follow‐up. European Journal of *Pain, 25*(5), 1012–1030. <https://doi.org/10.1002/ejp.1723>
43. Robles, E., Blanco, I., Díez, G., & Vázquez, C. (2024). Mindfulness‐based stress reduction for chronic pain: Enhancing psychological well‐being without altering attentional biases towards pain faces. *European Journal of Pain, 29*(2), e4714. <https://doi.org/10.1002/ejp.4714>
44. Roslyakova, T., Falco, M.-A., & Gauchet, A. (2020). An exploratory clinical trial on acceptance and commitment therapy as an adjunct to psychoeducational relaxation therapy for chronic pain. *Psychology & Health, 36*(12), 1403–1426. <https://doi.org/10.1080/08870446.2020.1856844>
45. Sanabria-Mazo, J. P., Colomer-Carbonell, A., Borràs, X., Castaño-Asins, J. R., McCracken, L. M., Montero-Marin, J., Pérez-Aranda, A., Edo, S., Sanz, A., Feliu-Soler, A., & Luciano, J. V. (2023). Efficacy of videoconference group acceptance and commitment therapy (ACT) and behavioral activation therapy for depression (BATD) for chronic low back pain (CLBP) plus comorbid depressive symptoms: A randomized controlled trial (IMPACT study). *The Journal of Pain, 24*(8), 1522–1540. <https://doi.org/10.1016/j.jpain.2023.04.008>
46. Scott, W., Chilcot, J., Guildford, B., Daly‐Eichenhardt, A., & McCracken, L. M. (2018). Feasibility randomized‐controlled trial of online acceptance and commitment therapy for patients with complex chronic pain in the United Kingdom. *European Journal of Pain, 22*(8), 1473–1484. <https://doi.org/10.1002/ejp.1236>
47. Scott, W., Guildford, B. J., Badenoch, J., Driscoll, E., Chilcot, J., Norton, S., Kemp, H. I., Lee, M. J., Lwanga, J., Boffito, M., Moyle, G., Post, F. A., Campbell, L., Josh, J., Clift, P., C De C Williams, A., Rice, A. S., & McCracken, L. M. (2020). Feasibility randomized‐controlled trial of online acceptance and commitment therapy for painful peripheral neuropathy in people living with HIV: The OPEN study. *European Journal of Pain, 25*(7), 1493–1507. <https://doi.org/10.1002/ejp.1762>
48. Simister, H. D., Tkachuk, G. A., Shay, B. L., Vincent, N., Pear, J. J., & Skrabek, R. Q. (2018). Randomized controlled trial of online acceptance and commitment therapy for fibromyalgia. *The Journal of Pain, 19*(7), 741–753. <https://doi.org/10.1016/j.jpain.2018.02.004>
49. Taheri, A. A., Foroughi, A. A., Mohammadian, Y., Ahmadi, S. M., Heshmati, K., Hezarkhani, L. A., & Parvizifard, A. A. (2020). The effectiveness of acceptance and commitment therapy on pain acceptance and pain perception in patients with painful diabetic neuropathy: A randomized controlled trial. *Diabetes Therapy, 11*(8), 1695–1708. <https://doi.org/10.1007/s13300-020-00851-9>
50. Taub, R., Agmon-Levin, N., Frumer, L., Samuel-Magal, I., Glick, I., & Horesh, D. (2024). Mindfulness-based stress reduction (MBSR) for fibromyalgia patients: The role of pain cognitions as mechanisms of change*. Complementary Therapies in Clinical Practice*, 56, 101860. <https://doi.org/10.1016/j.ctcp.2024.101860>
51. Thorsell, J., Finnes, A., Dahl, J., Lundgren, T., Gybrant, M., Gordh, T., & Buhrman, M. (2011). A comparative study of 2 manual-based self-help interventions, acceptance and commitment therapy and applied relaxation, for persons with chronic pain*. The Clinical Journal of Pain, 27*(8), 716–723. <https://doi.org/10.1097/AJP.0b013e318219a933>
52. Torrijos‐Zarcero, M., Mediavilla, R., Rodríguez‐Vega, B., Del Río‐Diéguez, M., López‐Álvarez, I., Rocamora‐González, C., & Palao‐Tarrero, Á. (2021). Mindful self‐compassion program for chronic pain patients: A randomized controlled trial. *European Journal of Pain, 25*(4), 930–944. <https://doi.org/10.1002/ejp.1734>
53. Trompetter, H. R., Bohlmeijer, E. T., Veehof, M. M., & Schreurs, K. M. G. (2015). Internet-based guided self-help intervention for chronic pain based on acceptance and commitment therapy: A randomized controlled trial. *Journal of Behavioral Medicine, 38*(1), 66–80. <https://doi.org/10.1007/s10865-014-9579-0>
54. Turner, J. A., Anderson, M. L., Balderson, B. H., Cook, A. J., Sherman, K. J., & Cherkin, D. C. (2016). Mindfulness-based stress reduction and cognitive behavioral therapy for chronic low back pain: Similar effects on mindfulness, catastrophizing, self-efficacy, and acceptance in a randomized controlled trial. *Pain, 157*(11), 2434–2444. <https://doi.org/10.1097/j.pain.0000000000000635>
55. Varallo, G., Cattivelli, R., Giusti, E. M., Landi, G., Spatola, C., Ruggiero, G. M., Franceschini, C., Tossani, E., Grandi, S., Capodaglio, P., & Castelnuovo, G. (2023). The efficacy of a brief acceptance-based group intervention in a sample of female patients with fibromyalgia and comorbid obesity: A randomised controlled trial. *Clinical and Experimental Rheumatology*. <https://doi.org/10.55563/clinexprheumatol/7hvaya>
56. Veillette, J., Martel, M.-E., & Dionne, F. (2019). A randomized controlled trial evaluating the effectiveness of an acceptance and commitment therapy–based bibliotherapy intervention among adults living with chronic pain. *Canadian Journal of Pain, 3*(1), 209–225. <https://doi.org/10.1080/24740527.2019.1678113>
57. Wetherell, J. L., Afari, N., Rutledge, T., Sorrell, J. T., Stoddard, J. A., Petkus, A. J., Solomon, B. C., Lehman, D. H., Liu, L., Lang, A. J., & Atkinson, H. J. (2011). A randomized, controlled trial of acceptance and commitment therapy and cognitive-behavioral therapy for chronic pain. *Pain, 152*(9), 2098–2107. <https://doi.org/10.1016/j.pain.2011.05.016>
58. Wicksell, R. K., Ahlqvist, J., Bring, A., Melin, L., & Olsson, G. L. (2008). Can exposure and acceptance strategies improve functioning and life satisfaction in people with chronic pain and whiplash‐associated disorders (WAD)? A randomized controlled trial. *Cognitive Behaviour Therapy, 37(*3), 169–182. <https://doi.org/10.1080/16506070802078970>
59. Wicksell, R. K., Kemani, M., Jensen, K., Kosek, E., Kadetoff, D., Sorjonen, K., Ingvar, M., & Olsson, G. L. (2013). Acceptance and commitment therapy for fibromyalgia: A randomized controlled trial. *European Journal of Pain, 17*(4), 599–611. <https://doi.org/10.1002/j.1532-2149.2012.00224.x>
60. Zgierska, A. E., Burzinski, C. A., Cox, J., Kloke, J., Stegner, A., Cook, D. B., ... & Bačkonja, M. (2016). Mindfulness meditation and cognitive behavioral therapy intervention reduces pain severity and sensitivity in opioid-treated chronic low back pain: pilot findings from a randomized controlled trial*. Pain Medicine, 17*(10), 1865-1881. <https://doi.org/10.1093/pm/pnw006>
