# Supplementary material for: Do Acceptance‐ and Mindfulness‐Based Interventions Improve Psychological Flexibility in People With Chronic Pain? A Systematic Review and Meta‐Analysis of Randomized Controlled Trials
Source: Eur J Pain. 2026 Aug 1;30(7):e70342. doi: 10.1002/ejp.70342 (PMC13428482; doi:10.1002/ejp.70342)

**Supplementary Material 3.** Eggers’ test and Funnel Plots.

|  | **Intercept** | **95% CI** | ***t*** | ***p*** |
| --- | --- | --- | --- | --- |
| **Global Flexibility**  **(*n* = 17)** | 0.71 | -1.45 to - 2.88 | 0.65 | 0.528 |
| **Acceptance**  **(*n=* 38)** | 2.12 | -0.38 to - 4.63 | 1.66 | 0.105 |
|  |  |  |  |  |

*Eggers' test does not indicate the presence of funnel plot asymmetry.


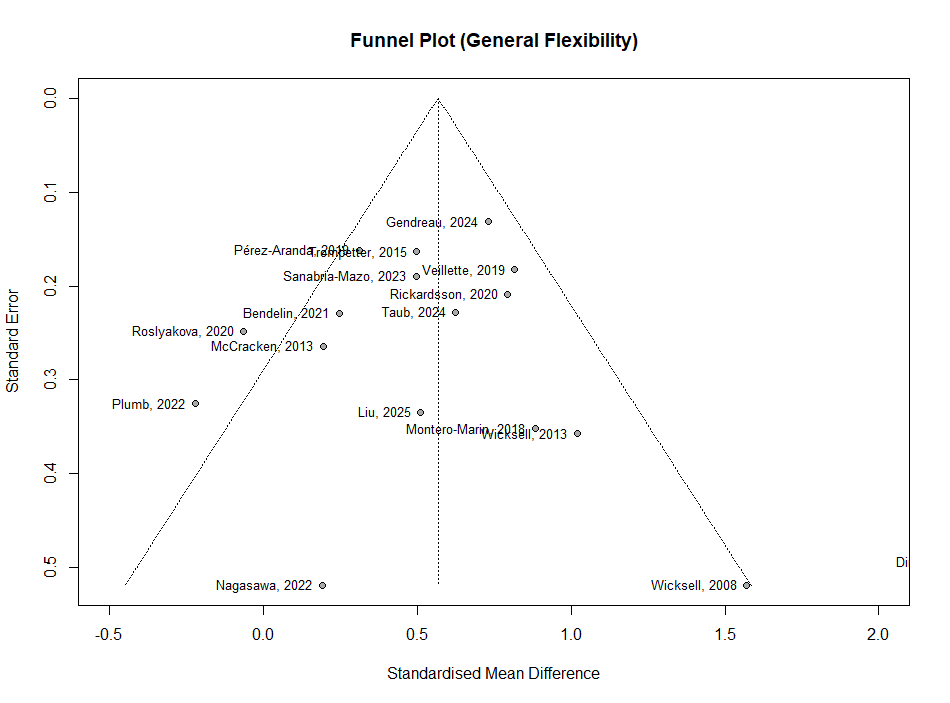


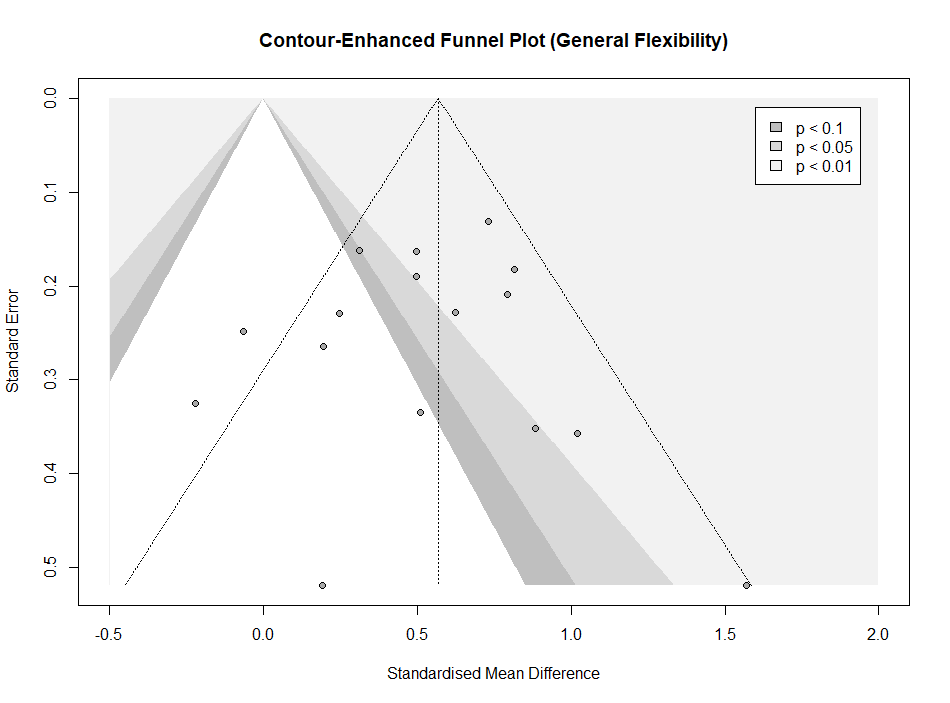


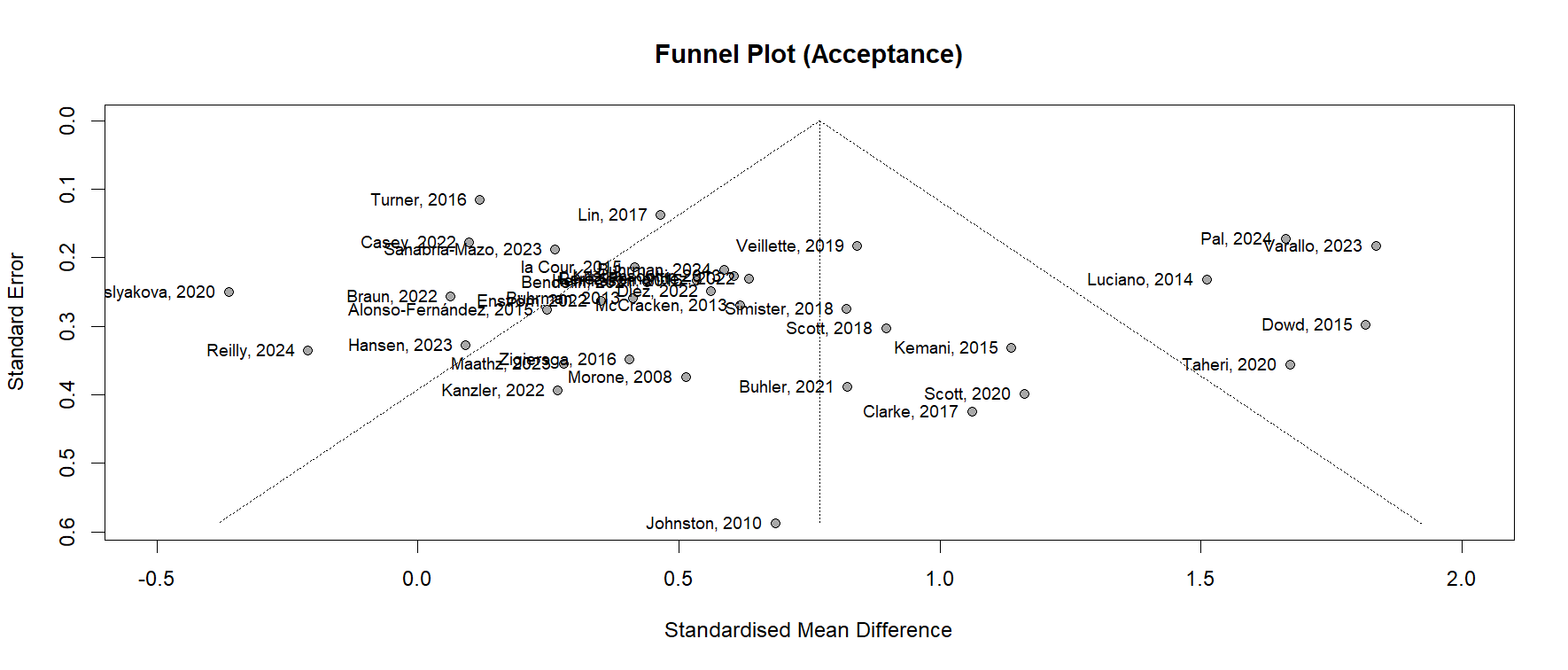


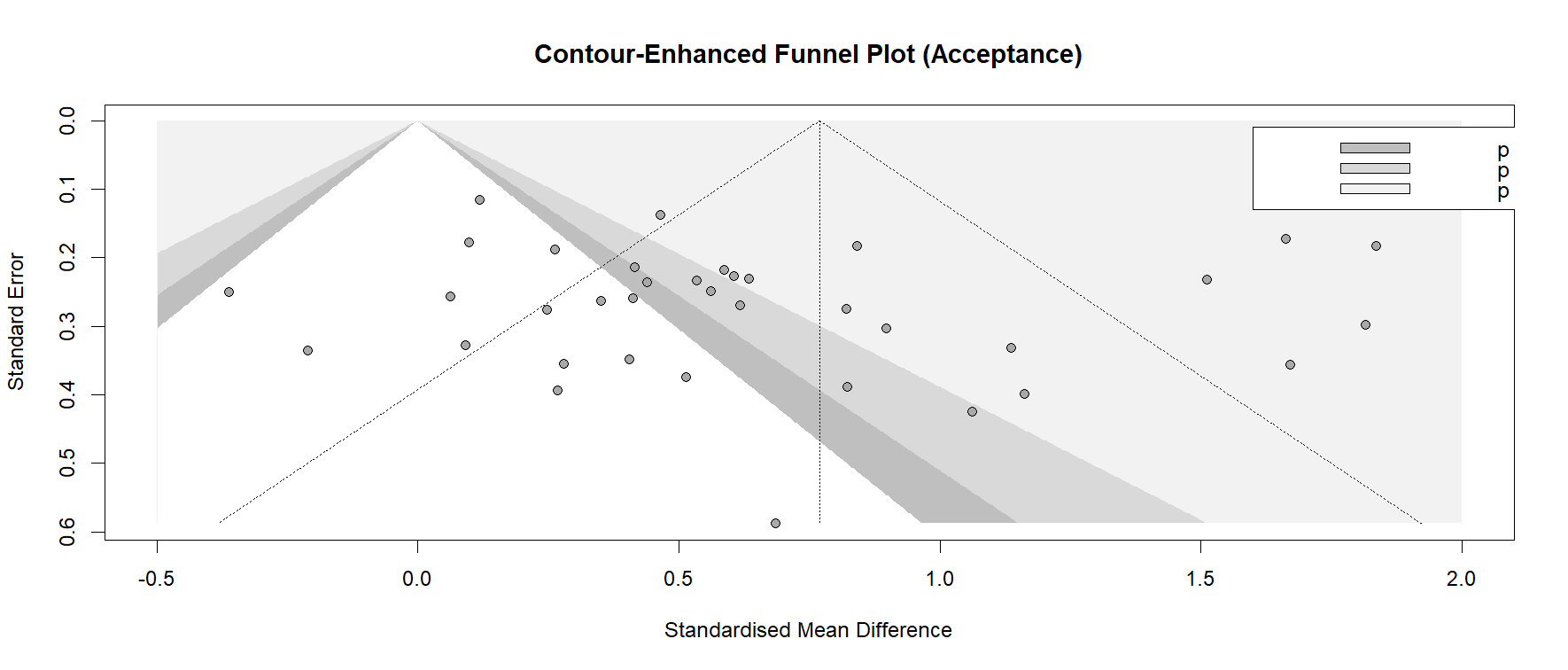

Supplement: Supplementary file 3 — Data S3: Eggers' test and funnel plots. [file EJP-30-0-s003.docx]
