# Supplementary material for: Do Acceptance‐ and Mindfulness‐Based Interventions Improve Psychological Flexibility in People With Chronic Pain? A Systematic Review and Meta‐Analysis of Randomized Controlled Trials
Source: Eur J Pain. 2026 Aug 1;30(7):e70342. doi: 10.1002/ejp.70342 (PMC13428482; doi:10.1002/ejp.70342)
Supplement: Supplementary file 4 — Table S1: Search strategy. [file EJP-30-0-s005.docx]

**Supplementary Table 1**. Search strategy

| **Database** | **Search strategy** |
| --- | --- |
| **Web of Science**  **(*n* = 724)** | (TI=("chronic pain" OR "chronic widespread pain" OR "chronic widespread pain syndrome" OR "persistent pain" OR "long-term pain" OR "chronic non-malignant pain" OR "low back pain" OR "back pain" OR "chronic musculoskeletal pain" OR "musculoskeletal pain" OR "fibromyalgia" OR "complex regional pain syndrome" OR "neuropathic pain" OR "chronic pelvic pain" OR "arthritis pain" OR "chronic joint pain" OR "chronic neck pain" OR "chronic shoulder pain" OR "chronic visceral pain" OR "central sensitization") OR AB=("chronic pain" OR "chronic widespread pain" OR "chronic widespread pain syndrome" OR "persistent pain" OR "long-term pain" OR "chronic non-malignant pain" OR "low back pain" OR "back pain" OR "chronic musculoskeletal pain" OR "musculoskeletal pain" OR "fibromyalgia" OR "complex regional pain syndrome" OR "neuropathic pain" OR "chronic pelvic pain" OR "arthritis pain" OR "chronic joint pain" OR "chronic neck pain" OR "chronic shoulder pain" OR "chronic visceral pain" OR "central sensitization") OR AK=("chronic pain" OR "chronic widespread pain" OR "chronic widespread pain syndrome" OR "persistent pain" OR "long-term pain" OR "chronic non-malignant pain" OR "low back pain" OR "back pain" OR "chronic musculoskeletal pain" OR "musculoskeletal pain" OR "fibromyalgia" OR "complex regional pain syndrome" OR "neuropathic pain" OR "chronic pelvic pain" OR "arthritis pain" OR "chronic joint pain" OR "chronic neck pain" OR "chronic shoulder pain" OR "chronic visceral pain" OR "central sensitization")) AND (TI=("cognitive behavioral therapy" OR "cognitive behaviour therapy" OR "CBT" OR "cognitive therapy" OR "third wave therapy" OR "contextual behavioral therapy" OR "acceptance and commitment therapy" OR "ACT" OR "acceptance-based therapy" OR "acceptance-based intervention" OR "mindfulness-based cognitive therapy" OR "MBCT" OR "mindfulness-based stress reduction" OR "MBSR" OR "mindfulness-based intervention" OR "dialectical behavior therapy" OR "dialectical behaviour therapy" OR "DBT" OR "behavioral activation therapy" OR "behavioural activation therapy" OR "metacognitive therapy" OR "MCT" OR "compassion-focused therapy" OR "CFT") OR AB=("cognitive behavioral therapy" OR "cognitive behaviour therapy" OR "CBT" OR "cognitive therapy" OR "third wave therapy" OR "contextual behavioral therapy" OR "acceptance and commitment therapy" OR "ACT" OR "acceptance-based therapy" OR "acceptance-based intervention" OR "mindfulness-based cognitive therapy" OR "MBCT" OR "mindfulness-based stress reduction" OR "MBSR" OR "mindfulness-based intervention" OR "dialectical behavior therapy" OR "dialectical behaviour therapy" OR "DBT" OR "behavioral activation therapy" OR "behavioural activation therapy" OR "metacognitive therapy" OR "MCT" OR "compassion-focused therapy" OR "CFT") OR AK=("cognitive behavioral therapy" OR "cognitive behaviour therapy" OR "CBT" OR "cognitive therapy" OR "third wave therapy" OR "contextual behavioral therapy" OR "acceptance and commitment therapy" OR "ACT" OR "acceptance-based therapy" OR "acceptance-based intervention" OR "mindfulness-based cognitive therapy" OR "MBCT" OR "mindfulness-based stress reduction" OR "MBSR" OR "mindfulness-based intervention" OR "dialectical behavior therapy" OR "dialectical behaviour therapy" OR "DBT" OR "behavioral activation therapy" OR "behavioural activation therapy" OR "metacognitive therapy" OR "MCT" OR "compassion-focused therapy" OR "CFT")) AND (TI=("psychological flexibility" OR "cognitive flexibility" OR "psychological inflexibility" OR "cognitive inflexibility" OR "emotional inflexibility" OR "acceptance" OR "experiential acceptance" OR "emotional acceptance" OR "defusion" OR "cognitive defusion" OR "self-as-context" OR "perspective taking" OR "present-moment awareness" OR "mindfulness" OR "values-based action" OR "personal values" OR "committed action" OR "goal-directed behavior" OR "avoidance" OR "experiential avoidance" OR "emotional avoidance" OR "fusion" OR "cognitive fusion" OR "self-as-content" OR "self-attachment" OR "cognitive distraction" OR "lack of values clarity" OR "values confusion" OR "behavioral avoidance" OR "avoidant behavior") OR AB=("psychological flexibility" OR "cognitive flexibility" OR "psychological inflexibility" OR "cognitive inflexibility" OR "emotional inflexibility" OR "acceptance" OR "experiential acceptance" OR "emotional acceptance" OR "defusion" OR "cognitive defusion" OR "self-as-context" OR "perspective taking" OR "present-moment awareness" OR "mindfulness" OR "values-based action" OR "personal values" OR "committed action" OR "goal-directed behavior" OR "avoidance" OR "experiential avoidance" OR "emotional avoidance" OR "fusion" OR "cognitive fusion" OR "self-as-content" OR "self-attachment" OR "cognitive distraction" OR "lack of values clarity" OR "values confusion" OR "behavioral avoidance" OR "avoidant behavior") OR AK=("psychological flexibility" OR "cognitive flexibility" OR "psychological inflexibility" OR "cognitive inflexibility" OR "emotional inflexibility" OR "acceptance" OR "experiential acceptance" OR "emotional acceptance" OR "defusion" OR "cognitive defusion" OR "self-as-context" OR "perspective taking" OR "present-moment awareness" OR "mindfulness" OR "values-based action" OR "personal values" OR "committed action" OR "goal-directed behavior" OR "avoidance" OR "experiential avoidance" OR "emotional avoidance" OR "fusion" OR "cognitive fusion" OR "self-as-content" OR "self-attachment" OR "cognitive distraction" OR "lack of values clarity" OR "values confusion" OR "behavioral avoidance" OR "avoidant behavior") OR ALL=("psychological flexibility" OR "cognitive flexibility" OR "psychological inflexibility" OR "cognitive inflexibility" OR "emotional inflexibility")) |
| **Scopus**  **(*n* = 644)** | (TITLE-ABS("chronic pain" OR "chronic widespread pain" OR "chronic widespread pain syndrome" OR "persistent pain" OR "long-term pain" OR "chronic non-malignant pain" OR "low back pain" OR "back pain" OR "chronic musculoskeletal pain" OR "musculoskeletal pain" OR "fibromyalgia" OR "complex regional pain syndrome" OR "neuropathic pain" OR "chronic pelvic pain" OR "arthritis pain" OR "chronic joint pain" OR "chronic neck pain" OR "chronic shoulder pain" OR "chronic visceral pain" OR "central sensitization") AND TITLE-ABS("cognitive behavioral therapy" OR "cognitive behaviour therapy" OR "CBT" OR "cognitive therapy" OR "third wave therapy" OR "contextual behavioral therapy" OR "acceptance and commitment therapy" OR "ACT" OR "acceptance-based therapy" OR "acceptance-based intervention" OR "mindfulness-based cognitive therapy" OR "MBCT" OR "mindfulness-based stress reduction" OR "MBSR" OR "mindfulness-based intervention" OR "dialectical behavior therapy" OR "dialectical behaviour therapy" OR "DBT" OR "behavioral activation therapy" OR "behavioural activation therapy" OR "metacognitive therapy" OR "MCT" OR "compassion-focused therapy" OR "CFT") AND TITLE-ABS("psychological flexibility" OR "cognitive flexibility" OR "psychological inflexibility" OR "cognitive inflexibility" OR "emotional inflexibility" OR "acceptance" OR "experiential acceptance" OR "emotional acceptance" OR "defusion" OR "cognitive defusion" OR "self-as-context" OR "perspective taking" OR "present-moment awareness" OR "mindfulness" OR "values-based action" OR "personal values" OR "committed action" OR "goal-directed behavior" OR "avoidance" OR "experiential avoidance" OR "emotional avoidance" OR "fusion" OR "cognitive fusion" OR "self-as-content" OR "self-attachment" OR "cognitive distraction" OR "lack of values clarity" OR "values confusion" OR "behavioral avoidance" OR "avoidant behavior")) |
| **Medline**  **(*n* = 735)** | (TI=("chronic pain" OR "chronic widespread pain" OR "chronic widespread pain syndrome" OR "persistent pain" OR "long-term pain" OR "chronic non-malignant pain" OR "low back pain" OR "back pain" OR "chronic musculoskeletal pain" OR "musculoskeletal pain" OR "fibromyalgia" OR "complex regional pain syndrome" OR "neuropathic pain" OR "chronic pelvic pain" OR "arthritis pain" OR "chronic joint pain" OR "chronic neck pain" OR "chronic shoulder pain" OR "chronic visceral pain" OR "central sensitization") OR AB=("chronic pain" OR "chronic widespread pain" OR "chronic widespread pain syndrome" OR "persistent pain" OR "long-term pain" OR "chronic non-malignant pain" OR "low back pain" OR "back pain" OR "chronic musculoskeletal pain" OR "musculoskeletal pain" OR "fibromyalgia" OR "complex regional pain syndrome" OR "neuropathic pain" OR "chronic pelvic pain" OR "arthritis pain" OR "chronic joint pain" OR "chronic neck pain" OR "chronic shoulder pain" OR "chronic visceral pain" OR "central sensitization") OR MH=("chronic pain" OR "chronic widespread pain" OR "chronic widespread pain syndrome" OR "persistent pain" OR "long-term pain" OR "chronic non-malignant pain" OR "low back pain" OR "back pain" OR "chronic musculoskeletal pain" OR "musculoskeletal pain" OR "fibromyalgia" OR "complex regional pain syndrome" OR "neuropathic pain" OR "chronic pelvic pain" OR "arthritis pain" OR "chronic joint pain" OR "chronic neck pain" OR "chronic shoulder pain" OR "chronic visceral pain" OR "central sensitization")) AND (TI=("cognitive behavioral therapy" OR "cognitive behaviour therapy" OR "CBT" OR "cognitive therapy" OR "third wave therapy" OR "contextual behavioral therapy" OR "acceptance and commitment therapy" OR "ACT" OR "acceptance-based therapy" OR "acceptance-based intervention" OR "mindfulness-based cognitive therapy" OR "MBCT" OR "mindfulness-based stress reduction" OR "MBSR" OR "mindfulness-based intervention" OR "dialectical behavior therapy" OR "dialectical behaviour therapy" OR "DBT" OR "behavioral activation therapy" OR "behavioural activation therapy" OR "metacognitive therapy" OR "MCT" OR "compassion-focused therapy" OR "CFT") OR AB=("cognitive behavioral therapy" OR "cognitive behaviour therapy" OR "CBT" OR "cognitive therapy" OR "third wave therapy" OR "contextual behavioral therapy" OR "acceptance and commitment therapy" OR "ACT" OR "acceptance-based therapy" OR "acceptance-based intervention" OR "mindfulness-based cognitive therapy" OR "MBCT" OR "mindfulness-based stress reduction" OR "MBSR" OR "mindfulness-based intervention" OR "dialectical behavior therapy" OR "dialectical behaviour therapy" OR "DBT" OR "behavioral activation therapy" OR "behavioural activation therapy" OR "metacognitive therapy" OR "MCT" OR "compassion-focused therapy" OR "CFT") OR MH=("cognitive behavioral therapy" OR "cognitive behaviour therapy" OR "CBT" OR "cognitive therapy" OR "third wave therapy" OR "contextual behavioral therapy" OR "acceptance and commitment therapy" OR "ACT" OR "acceptance-based therapy" OR "acceptance-based intervention" OR "mindfulness-based cognitive therapy" OR "MBCT" OR "mindfulness-based stress reduction" OR "MBSR" OR "mindfulness-based intervention" OR "dialectical behavior therapy" OR "dialectical behaviour therapy" OR "DBT" OR "behavioral activation therapy" OR "behavioural activation therapy" OR "metacognitive therapy" OR "MCT" OR "compassion-focused therapy" OR "CFT")) AND (TI=("psychological flexibility" OR "cognitive flexibility" OR "psychological inflexibility" OR "cognitive inflexibility" OR "emotional inflexibility" OR "acceptance" OR "experiential acceptance" OR "emotional acceptance" OR "defusion" OR "cognitive defusion" OR "self-as-context" OR "perspective taking" OR "present-moment awareness" OR "mindfulness" OR "values-based action" OR "personal values" OR "committed action" OR "goal-directed behavior" OR "avoidance" OR "experiential avoidance" OR "emotional avoidance" OR "fusion" OR "cognitive fusion" OR "self-as-content" OR "self-attachment" OR "cognitive distraction" OR "lack of values clarity" OR "values confusion" OR "behavioral avoidance" OR "avoidant behavior") OR AB=("psychological flexibility" OR "cognitive flexibility" OR "psychological inflexibility" OR "cognitive inflexibility" OR "emotional inflexibility" OR "acceptance" OR "experiential acceptance" OR "emotional acceptance" OR "defusion" OR "cognitive defusion" OR "self-as-context" OR "perspective taking" OR "present-moment awareness" OR "mindfulness" OR "values-based action" OR "personal values" OR "committed action" OR "goal-directed behavior" OR "avoidance" OR "experiential avoidance" OR "emotional avoidance" OR "fusion" OR "cognitive fusion" OR "self-as-content" OR "self-attachment" OR "cognitive distraction" OR "lack of values clarity" OR "values confusion" OR "behavioral avoidance" OR "avoidant behavior") OR MH=("psychological flexibility" OR "cognitive flexibility" OR "psychological inflexibility" OR "cognitive inflexibility" OR "emotional inflexibility" OR "acceptance" OR "experiential acceptance" OR "emotional acceptance" OR "defusion" OR "cognitive defusion" OR "self-as-context" OR "perspective taking" OR "present-moment awareness" OR "mindfulness" OR "values-based action" OR "personal values" OR "committed action" OR "goal-directed behavior" OR "avoidance" OR "experiential avoidance" OR "emotional avoidance" OR "fusion" OR "cognitive fusion" OR "self-as-content" OR "self-attachment" OR "cognitive distraction" OR "lack of values clarity" OR "values confusion" OR "behavioral avoidance" OR "avoidant behavior")) |
| **PsycINFO**  **(*n* = 314)** | ALL("chronic pain" OR "chronic widespread pain" OR "chronic widespread pain syndrome" OR "persistent pain" OR "long-term pain" OR "chronic non-malignant pain" OR "low back pain" OR "back pain" OR "chronic musculoskeletal pain" OR "musculoskeletal pain" OR "fibromyalgia" OR "complex regional pain syndrome" OR "neuropathic pain" OR "chronic pelvic pain" OR "arthritis pain" OR "chronic joint pain" OR "chronic neck pain" OR "chronic shoulder pain" OR "chronic visceral pain" OR "central sensitization") AND ALL("cognitive behavioral therapy" OR "cognitive behaviour therapy" OR "CBT" OR "cognitive therapy" OR "third wave therapy" OR "contextual behavioral therapy" OR "acceptance and commitment therapy" OR "ACT" OR "acceptance-based therapy" OR "acceptance-based intervention" OR "mindfulness-based cognitive therapy" OR "MBCT" OR "mindfulness-based stress reduction" OR "MBSR" OR "mindfulness-based intervention" OR "dialectical behavior therapy" OR "dialectical behaviour therapy" OR "DBT" OR "behavioral activation therapy" OR "behavioural activation therapy" OR "metacognitive therapy" OR "MCT" OR "compassion-focused therapy" OR "CFT") AND ALL("psychological flexibility" OR "cognitive flexibility" OR "psychological inflexibility" OR "cognitive inflexibility" OR "emotional inflexibility" OR "acceptance" OR "experiential acceptance" OR "emotional acceptance" OR "defusion" OR "cognitive defusion" OR "self-as-context" OR "perspective taking" OR "present-moment awareness" OR "mindfulness" OR "values-based action" OR "personal values" OR "committed action" OR "goal-directed behavior" OR "avoidance" OR "experiential avoidance" OR "emotional avoidance" OR "fusion" OR "cognitive fusion" OR "self-as-content" OR "self-attachment" OR "cognitive distraction" OR "lack of values clarity" OR "values confusion" OR "behavioral avoidance" OR "avoidant behavior") |
| **CENTRAL (*n* = 1111)** | ("chronic pain" OR "chronic widespread pain" OR "chronic widespread pain syndrome" OR "persistent pain" OR "long-term pain" OR "chronic non-malignant pain" OR "low back pain" OR "back pain" OR "chronic musculoskeletal pain" OR "musculoskeletal pain" OR "fibromyalgia" OR "complex regional pain syndrome" OR "neuropathic pain" OR "chronic pelvic pain" OR "arthritis pain" OR "chronic joint pain" OR "chronic neck pain" OR "chronic shoulder pain" OR "chronic visceral pain" OR "central sensitization") AND ("cognitive behavioral therapy" OR "cognitive behaviour therapy" OR "CBT" OR "cognitive therapy" OR "third wave therapy" OR "contextual behavioral therapy" OR "acceptance and commitment therapy" OR "ACT" OR "acceptance-based therapy" OR "acceptance-based intervention" OR "mindfulness-based cognitive therapy" OR "MBCT" OR "mindfulness-based stress reduction" OR "MBSR" OR "mindfulness-based intervention" OR "dialectical behavior therapy" OR "dialectical behaviour therapy" OR "DBT" OR "behavioral activation therapy" OR "behavioural activation therapy" OR "metacognitive therapy" OR "MCT" OR "compassion-focused therapy" OR "CFT") AND ("psychological flexibility" OR "cognitive flexibility" OR "psychological inflexibility" OR "cognitive inflexibility" OR "emotional inflexibility" OR "acceptance" OR "experiential acceptance" OR "emotional acceptance" OR "defusion" OR "cognitive defusion" OR "self-as-context" OR "perspective taking" OR "present-moment awareness" OR "mindfulness" OR "values-based action" OR "personal values" OR "committed action" OR "goal-directed behavior" OR "avoidance" OR "experiential avoidance" OR "emotional avoidance" OR "fusion" OR "cognitive fusion" OR "self-as-content" OR "self-attachment" OR "cognitive distraction" OR "lack of values clarity" OR "values confusion" OR "behavioral avoidance" OR "avoidant behavior") in Title Abstract Keyword |
