# Supplementary material for: Do Acceptance‐ and Mindfulness‐Based Interventions Improve Psychological Flexibility in People With Chronic Pain? A Systematic Review and Meta‐Analysis of Randomized Controlled Trials
Source: Eur J Pain. 2026 Aug 1;30(7):e70342. doi: 10.1002/ejp.70342 (PMC13428482; doi:10.1002/ejp.70342)
Supplement: Supplementary file 5 — Table S2: Characteristics of excluded studies. [file EJP-30-0-s004.docx]

**Supplementary Table 2.** Characteristics of excluded studies

| **Study (year)** | **Reason for exclusion** |
| --- | --- |
| Moore et al. (2000) | No assessment of psychological flexibility |
| Zautra et al. (2008) | No assessment of psychological flexibility |
| Fjorback et al. (2011) | Full text not available |
| Gaylord et al. (2011) | No assessment of psychological flexibility |
| Kristjánsdóttir et al. (2011) | Not a peer-reviewed journal article |
| Schmidt et al. (2011) | No assessment of psychological flexibility |
| Olsson et al. (2012) | Not a peer-reviewed journal article |
| Brown & Jones (2013) | No assessment of psychological flexibility |
| Steiner et al. (2013) | No assessment of psychological flexibility |
| Thorsell et al. (2013) | Publication type not eligible (erratum) |
| Ussher et al. (2014) | Insufficient statistical data |
| Brotto et al. (2015) | Not a randomized controlled trial |
| Cash et al. (2015) | No assessment of psychological flexibility |
| Davis et al. (2015) | No assessment of psychological flexibility |
| Trompetter et al. (2015) | Insufficient statistical data |
| McGeary et al. (2016) | No assessment of psychological flexibility |
| Morone et al. (2016) | No assessment of psychological flexibility |
| Steurer (2016) | Full text not available |
| Nathan et al. (2017) | No assessment of psychological flexibility |
| Boersma et al. (2019) | No assessment of psychological flexibility |
| Critchley et al. (2019) | Not a peer-reviewed journal article |
| Howarth et al. (2019) | No assessment of psychological flexibility |
| Torrijos-Zarcero et al. (2019) | Not a peer-reviewed journal article |
| Hruschak et al. (2021) | No assessment of psychological flexibility |
| Thompson et al. (2021) | Not a peer-reviewed journal article |
| Amutio et al. (2022) | No assessment of psychological flexibility |
| Böhmer et al. (2022) | Not a CBT-based intervention |
| Mittal et al. (2022) | No assessment of psychological flexibility |
| Buhrman et al. (2023) | Not a peer-reviewed journal article |
| Burns et al. (2023) | No assessment of psychological flexibility |
| Casey et al. (2023) | Secondary publication of the included trial |
| Chen et al. (2023) | No assessment of psychological flexibility |
| Crisp et al. (2023) | No assessment of psychological flexibility |
| Deegan et al. (2023) | Not a peer-reviewed journal article |
| Garcia et al. (2023) | Population with pain secondary to a structural condition |
| Hanley & Lingard (2023) | Not a peer-reviewed journal article |
| Bilal & Altaf (2024) | Not a peer-reviewed journal article |
| Hanley et al. (2024) | Full text not available |
| Rabinowitz et al. (2024) | Population with pain secondary to a structural condition |
| Serrat et al. (2024) | No assessment of psychological flexibility |

*Note.* Studies are ordered chronologically by year of publication (oldest to most recent); within the same year, studies are listed alphabetically by first author.

**References**

Amutio, A., Franco, C., Soriano-Ayala, E., & Van Gordon, W. (2022). Flow meditation improves emotion regulation and pain management in female fibromyalgia patients. *Mindfulness*, *13*(10), 2587–2599. https://doi.org/10.1007/s12671-022-01981-w

Bilal, A., & Altaf, S. (2024). (053) Efficacy of mindfulness based cognitive therapy in management of provoked vulvodynia: A feasibility pilot study. *The Journal of Sexual Medicine*, *21*(Supplement_2), qdae002.049. https://doi.org/10.1093/jsxmed/qdae002.049

Boersma, K., Södermark, M., Hesser, H., Flink, I. K., Gerdle, B., & Linton, S. J. (2019). Efficacy of a transdiagnostic emotion–focused exposure treatment for chronic pain patients with comorbid anxiety and depression: A randomized controlled trial. *Pain*, *160*(8), 1708–1718. https://doi.org/10.1097/j.pain.0000000000001575

Böhmer, M. C., La Cour, P., & Schnell, T. (2022). A randomized controlled trial of the sources of meaning card method: A new meaning-oriented approach predicts depression, anxiety, pain acceptance, and crisis of meaning in patients with chronic pain. *Pain Medicine*, *23*(2), 314–325. https://doi.org/10.1093/pm/pnab321

Brotto, L. A., Basson, R., Smith, K. B., Driscoll, M., & Sadownik, L. (2015). Mindfulness-based group therapy for women with provoked vestibulodynia. *Mindfulness*, *6*(3), 417–432. https://doi.org/10.1007/s12671-013-0273-z

Brown, C. A., & Jones, A. K. P. (2013). Psychobiological correlates of improved mental health in patients with musculoskeletal pain after a mindfulness-based pain management program. *The Clinical Journal of Pain*, *29*(3), 233–244. https://doi.org/10.1097/AJP.0b013e31824c5d9f

Buhrman, M., Tillfors, M., Holländare, F., Lekström, E., Håkansson, A., & Boersma, K. (2023). Psychological treatment targeting acceptance and compassion in patients with chronic pain: A randomized controlled, internet-delivered, treatment trial. *The Clinical Journal of Pain*, *39*(12), 672–685. https://doi.org/10.1097/AJP.0000000000001157

Burns, J. W., Jensen, M. P., Gerhart, J., Thorn, B. E., Lillis, T. A., Carmody, J., & Keefe, F. (2023). Cognitive therapy, mindfulness-based stress reduction, and behavior therapy for people with chronic low back pain: A comparative mechanisms study. *Journal of Consulting and Clinical Psychology*, *91*(3), 171–187. https://doi.org/10.1037/ccp0000801

Casey, M., Takemasa, S., Fullen, B., O’reilly, T., Leamy, M., Mc Kearney, E., Buckley, M., Smart, K., Hearty, C., & Doody, C. (2023). Exercise combined with acceptance and commitment therapy for adults with chronic pain: A randomized controlled trial one year follow-up. *Health Professionals in Rheumatology Abstracts*, 249–249. https://doi.org/10.1136/annrheumdis-2023-eular.2045

Cash, E., Salmon, P., Weissbecker, I., Rebholz, W. N., Bayley-Veloso, R., Zimmaro, L. A., Floyd, A., Dedert, E., & Sephton, S. E. (2015). Mindfulness meditation alleviates fibromyalgia symptoms in women: Results of a randomized clinical trial. *Annals of Behavioral Medicine*, *49*(3), 319–330. https://doi.org/10.1007/s12160-014-9665-0

Chen, S., Gao, X., Shi, T., Zuo, X., Hong, C., Zhang, Y., You, B., Li, F., Jackson, T., & He, Y. (2023). Promising subjective and objective benefits of modified mindfulness-based stress reduction training for Chinese adults with chronic rain: A pilot randomized control study. *Pain and Therapy*, *12*(6), 1397–1414. https://doi.org/10.1007/s40122-023-00551-9

Crisp, C. D., Baldi, R., Fuller, M., Abreu, E., & Nackley, A. G. (2023). Complementary approaches for military women with chronic pelvic pain: A randomized trial. *Journal of Integrative and Complementary Medicine*, *29*(1), 22–30. https://doi.org/10.1089/jicm.2022.0616

Critchley, D., McCracken, L., Wileman, V., Galea Holmes, M., Norton, S., & Godfrey, E. (2019). Physiotherapy informed by acceptance and commitment therapy (PACT) for people with chronic low back pain: A randomised controlled trial. *Physiotherapy*, *105*, e34–e35. https://doi.org/10.1016/j.physio.2018.11.279

Davis, M. C., Zautra, A. J., Wolf, L. D., Tennen, H., & Yeung, E. W. (2015). Mindfulness and cognitive–behavioral interventions for chronic pain: Differential effects on daily pain reactivity and stress reactivity. *Journal of Consulting and Clinical Psychology*, *83*(1), 24–35. https://doi.org/10.1037/a0038200

Deegan, O., Fullen, B. M., Casey, M.-B., Segurado, R., Hearty, C., & Doody, C. (2023). Mindfulness combined with exercise online (MOVE) compared with a self-management guide for adults with chronic pain: A feasibility randomized controlled trial. *The Clinical Journal of Pain*, *39*(8), 394–407. https://doi.org/10.1097/AJP.0000000000001126

Fjorback, L., Schröder, A., Ørnbøl, E., Rehfeld, E., Arendt, M., & Fink, P. (2011). XIV Annual Meeting of the European Association for Consultation Liaison Psychiatry and Psychosomatics (EACLPP). *Journal of Psychosomatic Research*, *70*(6), 580–623. https://doi.org/10.1016/j.jpsychores.2011.03.006

Garcia, M. A., Rabinowitz, E. P., Levin, M. E., Shasteen, H., Allen, P. A., & Delahanty, D. L. (2023). Online acceptance and commitment therapy for chronic pain in a sample of people with Chiari malformation: A pilot study. *Journal of Behavioral and Cognitive Therapy*, *33*(3), 152–168. https://doi.org/10.1016/j.jbct.2023.09.001

Gaylord, S., Palsson, O. S., Garland, E., Faurot, K., Coble, R. S., Mann, D., & Whitehead, W. E. (2011). Therapeutic impact of mindfulness meditation on irritable bowel syndrome (IBS): Results of a randomized controlled trial. *Gastroenterology*, *140*(5), S-50. https://doi.org/10.1016/S0016-5085(11)60203-5

Hanley, A., & Lingard, A. (2023). A single-session, two-hour mindfulness intervention improved chronic pain-related outcomes three months later. *The Journal of Pain*, *24*(4), 47. https://doi.org/10.1016/j.jpain.2023.02.144

Hanley, A. W., Lingard, A., & Garland, E. L. (2024). A single-session, 2-hour version of mindfulness-oriented recovery enhancement (One MORE) improves chronic pain patients’ pain-related outcomes through 3-month follow-up in a randomized controlled trial. *Journal of Integrative and Complementary Medicine*, *30*(9), 869–877. https://doi.org/10.1089/jicm.2023.0501

Howarth, A., Riaz, M., Perkins-Porras, L., Smith, J. G., Subramaniam, J., Copland, C., Hurley, M., Beith, I., & Ussher, M. (2019). Pilot randomised controlled trial of a brief mindfulness-based intervention for those with persistent pain. *Journal of Behavioral Medicine*, *42*(6), 999–1014. https://doi.org/10.1007/s10865-019-00040-5

Hruschak, V., Rosen, D., Tierney, M., Eack, S. M., Wasan, A. D., & Cochran, G. (2021). Integrated psychosocial group treatment: A randomized pilot trial of a harm reduction and preventive approach for patients with chronic pain at risk of opioid misuse. *Pain Medicine*, *22*(9), 2007–2018. https://doi.org/10.1093/pm/pnaa461

Kristjánsdóttir, Ó. B., Fors, E. A., Eide, E., Finset, A., Van Dulmen, S., Wigers, S. H., & Eide, H. (2011). The effect of web-based diaries and situational feedback on catastrophizing in women with chronic widespread pain: A randomized trial. *European Journal of Pain Supplements*, *5*(S1), 259–260. https://doi.org/10.1016/S1754-3207(11)70897-5

McGeary, C. A., Blount, T. H., Peterson, A. L., Gatchel, R. J., Hale, W. J., & McGeary, D. D. (2016). Interpersonal responses and pain management within the US military. *Journal of Occupational Rehabilitation*, *26*(2), 216–228. https://doi.org/10.1007/s10926-015-9605-2

Mittal, T. K., Evans, E., Pottle, A., Lambropoulos, C., Morris, C., Surawy, C., Chuter, A., Cox, F., De Silva, R., Mason, M., Banya, W., Thakrar, D., & Tyrer, P. (2022). Mindfulness-based intervention in patients with persistent pain in chest (MIPIC) of non-cardiac cause: A feasibility randomised control study. *Open Heart*, *9*(1), e001970. https://doi.org/10.1136/openhrt-2022-001970

Moore, J. E., Von Korff, M., Cherkin, D., Saunders, K., & Lorig, K. (2000). A randomized trial of a cognitive-behavioral program for enhancing back pain self care in a primary care setting. *Pain*, *88*(2), 145–153. https://doi.org/10.1016/S0304-3959(00)00314-6

Morone, N. E., Greco, C. M., Moore, C. G., Rollman, B. L., Lane, B., Morrow, L. A., Glynn, N. W., & Weiner, D. K. (2016). A mind-body program for older adults with chronic low back pain: A randomized clinical trial. *JAMA Internal Medicine*, *176*(3), 329. https://doi.org/10.1001/jamainternmed.2015.8033

Nathan, H. J., Poulin, P., Wozny, D., Taljaard, M., Smyth, C., Gilron, I., Sorisky, A., Lochnan, H., & Shergill, Y. (2017). Randomized trial of the effect of mindfulness-based stress reduction on pain-related disability, pain intensity, health-related quality of life, and A1C in patients with painful diabetic peripheral neuropathy. *Clinical Diabetes*, *35*(5), 294–304. https://doi.org/10.2337/cd17-0077

Olsson, G. L., Kemani, M., Jensen, K., Kosek, E., Kadetoff, D., Sorjonen, K., Ingvar, M., & Wicksell, R. (2012). Acceptance and commitment therapy for fibromyalgia: A randomized controlled trial. *Scandinavian Journal of Pain*, *3*(3), 183–183. https://doi.org/10.1016/j.sjpain.2012.05.027

Rabinowitz, E. P., Ripley, G., Levin, M. E., Allen, P. A., & Delahanty, D. L. (2024). Limited effects of phone coaching in an RCT of online self-guided acceptance and commitment therapy for chronic pain. *Journal of Contextual Behavioral Science*, *34*, 100828. https://doi.org/10.1016/j.jcbs.2024.100828

Schmidt, S., Grossman, P., Schwarzer, B., Jena, S., Naumann, J., & Walach, H. (2011). Treating fibromyalgia with mindfulness-based stress reduction: Results from a 3-armed randomized controlled trial. *Pain*, *152*(2), 361–369. https://doi.org/10.1016/j.pain.2010.10.043

Serrat, M., Navarrete, J., Ferrés, S., Auer, W., Sanmartín-Sentañes, R., Nieto, R., Neblett, R., Borràs, X., Luciano, J. V., & Feliu-Soler, A. (2024). Effectiveness of an online multicomponent program (FATIGUEWALK) for chronic fatigue syndrome: A randomized controlled trial. *Health Psychology*, *43*(4), 310–322. https://doi.org/10.1037/hea0001346

Steiner, J. L., Bogusch, L., & Bigatti, S. M. (2013). Values-based action in fibromyalgia: Results from a randomized pilot of acceptance and commitment therapy. *Health Psychology Research*, *1*(3), 34. https://doi.org/10.4081/hpr.2013.1542

Steurer, J. (2016). Mindfulness-based stress reduction is effective in patients with chronic lumbar backache. *Praxis*, *105*(12), 721–722. https://doi.org/10.1024/1661-8157/a002378

Thompson, J., Parikh, N., Gavigan, K., Venkatachalam, S., & Nowell, W. B. (2021). A mindfulness program dosing study to evaluate improvement in emotional distress among people with rheumatic disease. *Annals of the Rheumatic Diseases*, *80*, 169–170. https://doi.org/10.1136/annrheumdis-2021-eular.2728

Thorsell, J., Finnes, A., Dahl, J., Lundgren, T., Gybrant, M., Gordh, T., & Buhrman, M. (2013). A comparative study of 2 manual-based self-help interventions, acceptance and commitment therapy and applied relaxation, for persons with cchronic pain: Erratum. *The Clinical Journal of Pain*, *29*(5), 469. https://doi.org/10.1097/AJP.0b013e31829261d6

Torrijos-Zarcero, M., Palao-Tarrero, A., Rodriguez-Vega, B., Rocamora-Gonzalez, C., Del Rio, M., Calle, D., & Nocete, L. (2019). Randomized clinical trial to compare the effectiveness of a mindful self-compassion program and a behavioral-cognitive intervention to improve quality of life in chronic pain patients. *European Psychiatry*, *56*(S1), S3–S321. https://doi.org/10.1016/j.eurpsy.2019.01.003

Trompetter, H. R., Bohlmeijer, E. T., Fox, J.-P., & Schreurs, K. M. G. (2015). Psychological flexibility and catastrophizing as associated change mechanisms during online acceptance & commitment therapy for chronic pain. *Behaviour Research and Therapy*, *74*, 50–59. https://doi.org/10.1016/j.brat.2015.09.001

Ussher, M., Spatz, A., Copland, C., Nicolaou, A., Cargill, A., Amini-Tabrizi, N., & McCracken, L. M. (2014). Immediate effects of a brief mindfulness-based body scan on patients with chronic pain. *Journal of Behavioral Medicine*, *37*(1), 127–134. https://doi.org/10.1007/s10865-012-9466-5

Zautra, A. J., Davis, M. C., Reich, J. W., Nicassario, P., Tennen, H., Finan, P., Kratz, A., Parrish, B., & Irwin, M. R. (2008). Comparison of cognitive behavioral and mindfulness meditation interventions on adaptation to rheumatoid arthritis for patients with and without history of recurrent depression. *Journal of Consulting and Clinical Psychology*, *76*(3), 408–421. https://doi.org/10.1037/0022-006X.76.3.408
